# Removal of CD276^+^ cells from haploidentical memory T-cell grafts significantly lowers the risk of GVHD

**DOI:** 10.1038/s41409-021-01307-9

**Published:** 2021-05-11

**Authors:** Hisayoshi Hashimoto, Patrick Kasteleiner, Jakob Kressin, Friederike Müller, Hans-Jörg Bühring, Rupert Handgretinger, Karin Schilbach

**Affiliations:** 1 Department of Hematology and Oncology, Children’s Hospital, Universität Tübingen, Tübingen, Germany; 2 Department of General and Molecular Pathology and Pathological Anatomy, Institute of Pathology and Neuropathology, Universität Tübingen, Tübingen, Germany; 3 Department of Internal Medicine, University Hospital Tübingen, Tübingen, Germany

**Keywords:** Allotransplantation, Haematopoietic stem cells

## Abstract

Detrimental graft-versus-host disease (GVHD) still remains a major cause of death in hematopoietic stem cell transplantation (HSCT). The recently explored depletion of naive cells from mobilized grafts (CD45RA depletion) has shown considerable promise, yet is unable to eliminate the incidence of GVHD. Analysis of CD45RA-depleted haploidentical mixed lymphocytes culture (haplo-MLC) revealed insufficient suppression of alloresponses in the CD4^+^ compartment and identified CD276 as a marker for alloreactive memory Th1 T cells. Conclusively, depleting CD276^+^ cells from CD45RA-depleted haplo-MLC significantly attenuated alloreactivity to recipient cells while increasing antiviral reactivity and maintaining anti-third party reactivity in vitro. To evaluate these findings in vivo, bulk, CD45RA-depleted, or CD45RA/CD276-depleted CD4^+^ T cells from HLA-DR4^negative^ healthy humans were transplanted into NSG-Ab°DR4 mice, a sensitive human allo-GVHD model. Compellingly, CD45RA/CD276-depleted grafts from HLA-DR4^negative^ donors or in vivo depletion of CD276^+^ cells after transplant of HLA-DR4^negative^ memory CD4 T cells significantly delay the onset of GVHD symptoms and significantly alleviate its severity in NSG-Ab°DR4 mice. The clinical courses correlated with diminished Th1-cytokine secretion and downregulated CXCR6 expression of engrafted peripheral T cells. Collectively, mismatched HLA-mediated GVHD can be controlled by depleting recipient-specific CD276^+^ alloreacting T cells from the graft, highlighting its application in haplo-HSCT.

## Introduction

Hematopoietic stem cell transplantation (HSCT) is a standard therapy for high-risk hematologic malignancies. Since less than 30% of the patients in need for a transplant have a human leukocyte antigen (HLA)-identical sibling, it is imperative to have an alternative stem cell source such as a haploidentical donor when a matched donor cannot be obtained. The high HLA disparity in haploidentical transplantation (haplo-HSCT) demands intense prophylaxis and treatment to suppress donor-derived alloreacting T cells that are critical mediators of graft-versus-host disease (GVHD), responding to host major and minor histocompatibility complex-derived epitopes. On the other hand, prevention of relapse of the underlying disease is dependent on the graft-versus-tumor/leukemia (GVT/L) effect mediated by donor T cells. Therefore, to minimize the risk of GVHD while preserving the GVT/L effect and immunity against pathogens is a major therapeutic challenge in HSCT.

A recently investigated technique is the elimination of naive T cells from the allograft, following the rationale that naive T cells constitute the major source of potentially alloreactive precursors with their most diverse TCR repertoire [1]. A previous study showed that memory T cells can selectively be enriched by CD45RA depletion and that these memory T cells mediate significantly reduced alloreactivity in vitro, yet enhanced antitumor and antiviral response [2]. Multiple groups then demonstrated that although CD45RA-depleted haplo transplant is feasible [2–7], GVHD still evolves [3]. Thus, identification and characterization of residual antigen-experienced memory T-cell compartment that triggers GVHD in CD45RA-depleted HSCT is of special importance.

Recent accumulating evidence indicates a crucial role of the costimulatory/coinhibitory signals in regulating T-cell activities involving GVHD. For example, blockade of the CD40-CD40L and CD80/CD86-CD28/CTLA-4 (cytotoxic T-lymphocyte-associated protein 4) axes have been attempted to induce alloantigen specific T-cell energy and tolerance [8, 9]. Inducible T-cell costimulator (ICOS) plays an important role in inducing aGVHD and cGVHD [10, 11]. CD26^+^ T cells accumulate in GVHD target organs [12]. 4-1BB (CD137) was reported to be useful for identifying alloantigen-reactive T cells [13]. PD-1/PD-L1 blockade accelerates aGVHD via T_H_1 skewing, and PD-L1 deficiency exacerbates cGVHD [14]. B7-H3 (CD276) negatively regulates T-cell-mediated aGVHD in an MHC mismatched setting [15] and serum level of CD276 can predict severe aGVHD [16]. The blockade of the lymphocyte-activated gene-3 (LAG-3) signaling prevented murine GVHD [17]. The T-cell immunoglobulin and mucin-containing protein 3 (TIM-3) negatively regulates T_H_1 and T_c_1 responses and serum concentration of TIM-3 is positively correlated with the severity of aGVHD [18]. For these reasons, we investigated costimulatory/coinhibitory molecules as well as activation, proliferation, and memory phenotype markers expressed on alloreactive memory T cells in the setting of haplo-HSCT. The purpose of this study was to identify a molecule that uniquely defines alloreactive T cells in CD45RA-depleted grafts and to determine whether depletion from the graft of T cells which express this molecule after short term ex vivo mixed lymphocytes culture (MLC) with recipient cells can ameliorate GVHD.

## Materials and methods

### Depletion of CD45RA^+^ and CD276^+^ T cells

PBMCs were depleted of CD45RA^+^ cells by labeling with CliniMACS CD45RA Reagent (Miltenyi Biotech, Germany). Cells were then stained with anti-CD276-PE antibody (Miltenyi Biotech) and depleted using anti-PE MicroBeads and LD-depletion columns. Purity of CD45RA-/CD276-depleted cells was always >99%.

### Haploidentical MLC (haplo-MLC)

PBMCs from healthy adults aged 24–40 (responder/donor) and 24–70 years (stimulator/recipient) were cultured in RPMI 1640, 100 U/ml penicillin, 100 μg/ml streptomycin, 2 mM L-Glutamine, and 10% FBS. For CD45RA-depleted haplo-MLC, CD45RA-depleted PBMCs were stimulated in MLC at 1:1 ratio with irradiated (80 Gy) haploidentical PBMCs (1 × 10^6^ cells/ml in 24-well plates, respectively). 50 IU/ml rhIL-2, 1 ng/ml rhIL-7, and 10 ng/ml rhIL-15 were added on days 0 and 4. Responder cells were tested at day 7 by flow cytometry to determine their antigen-specific reactivity. In CD45RA/CD276-depleted haplo-MLC, CD276^+^ cells were depleted from CD45RA-depleted haplo-MLC on day 4. The cytokines described above were added on days 0, 4, and 7. After restimulation with irradiated allogeneic PBMCs (1:1) on day 4, responder cells were tested on day 11 by flow cytometry. Viabilities of 80 Gy-irradiated lymphocytes were 0% on day 7 and 2.74 ± 0.43% on day 4 in our study.

### CFSE proliferation assay

One micromolar CFSE solution (Life Technologies) was added to responder cells and CFSE-labeled cells then cocultured in a 1:1 ratio with irradiated target cells in 48-well plates for 4 days. Proliferation was then accessed by flow cytometry. 80 Gy-irradiated autologous PBMCs as targets allowed identification of background proliferation, 80 Gy-irradiated haploidentical stimulator PBMCs gave a measure for alloreacting cells.

### Testing for antiviral activity

Cultures were stimulated individually for 4 h with one of the peptides mixes (15-mer peptides with 11 amino acid overlap, covering the complete sequence of CMV pp65, EBV consensus, or AdV5 antigens (Miltenyi Biotec)) in 96-well plates. Simultaneously, brefeldin A (BioLegend) was added and cultures were analyzed 4 h later by flow cytometry to determine reactivity and antigen specificity.

### Isolation of CD3^+^CD276^+^ cells and CD3^+^CD276^−^ cells in haplo-MLC

CD3^+^ cells were isolated using CD3-FITC (BD Biosciences) and anti-FITC MultiSort kit (Miltenyi Biotec). CD276^−^ or CD276^+^ cells were then isolated with LD or MS column, after staining with CD276-PE and anti-PE MicroBeads (Miltenyi Biotec). Purity: CD3^+^ > 97%, CD276^−^ > 98.5%, and CD276^+^ > 96%.

### RNA extraction, cDNA synthesis

RNA was extracted with the RNeasy Mini Kit (Qiagen, Hilden, Germany) and reverse transcription using Superscript III First Strand Synthesis Super Mix (Life Technology, Germany).

### Real-time PCR

Expression of Eomes, T-bet, Gata3, Foxp3, RORC, IFN-γ, and GAPDH was analyzed with qPCR and SYBR Green (Promega, USA) in a Bio-Rad C1000 Thermal cycler/CFX96 real-time System (Bio-Rad, Germany). Primers are listed in Supplementary Table 1.

### Flow cytometric analysis

Antibodies are listed in Supplementary Table 2. For intracellular stainings, BD Cytofix/Cytoperm kit (BD Biosciences) was used. For IL-17A staining, cells were cultured at 37 °C for 4 h in 96-well plates in 200 μl culture medium in the presence of 0.2 μl brefeldin A, 50 ng/ml phorbol 12-myristate 13-acetate (PMA, Sigma-Aldrich), plus 500 ng/ml ionomycin (Sigma-Aldrich). For IFN-γ and TNF-α stainings cells were incubated with brefeldin A and monensin (BD Biosciences) for 4 h. Dead cells were excluded with Zombie Aqua Fixable viability kit (BioLegend). Samples were analyzed on an LSR Fortessa or FACS Canto II (BD Biosciences) using fluorescence minus one control. Subsequent analyses were done with FlowJo software (TreeStar, Inc., USA).

### Mice

Twelve to twenty four weeks old female NOD.Cg-PrkdcscidIl2rgtm1WjlH2-Ab1tm1GruTg(HLA-DRB1)31Dmz/SzJ (NSG-Ab°DR4) mice were purchased from the Jackson Laboratory and housed in single airflow cages under specific pathogen-free conditions. Animal experiments were according to the standards of the ethic committee board and animal care committee of the University of Tübingen, the approval numbers were: K01/17, K05/18, and K04/19G.

### Transplantation of human CD4^+^ T cells to NSG-Ab°DR4 mice

NSG-Ab°DR4 mice received a single injection in the tail vein with one of the following three different grafts, respectively (4 × 10^6^ cells/mouse): HLA-DR4^negative^ or HLA-DR4^positive^ donor’s (A) bulk CD4^+^ T cells, or (B) CD45RA-depleted memory CD4^+^ T cells, or (C) CD45RA/CD276-depleted memory CD4^+^ T cells. Individuals were scored daily for five parameters of clinical GVHD (posture, activity, fur, skin, and weight loss) on a scale from 0 to 2. Clinical GVHD score was assessed by summation of these parameters as reported [19]. Mice were sacrificed when reaching a single score of 1.5 or exceeding clinical score of 4 or when reaching day 100. Engraftment was defined as >5% human cells in whole PBMCs. If the engraftment was not achieved by day 35, mice were excluded from the analysis. No randomization and no blinding were conducted. Sample size was estimated based on preliminary experiments.

### Isolation of bulk CD4^+^, memory CD4^+^, and CD276^−^ memory CD4^+^ T cells for transplantation

CD4^+^ T-cell isolation kit and Memory CD4^+^ T-cell isolation kit (Miltenyi Biotech) were used for isolating bulk CD4^+^ T cells and CD45RA-memory CD4^+^ T cells, respectively, according to the manufacture’s instruction. Purity: CD4^+^ > 97%, CD45RA-CD4^+^ > 99%. Then, isolated memory CD4^+^ T cells were stimulated in MLC at 3:1 ratio with irradiated (80 Gy) DR4 mouse PBMCs (1.2 × 10^6^ cells/ml and 4 × 10^5^ cells/ml in 48-well plates, respectively). 50 IU/ml rhIL-2, 1 ng/ml rhIL-7, and 10 ng/ml rhIL-15 were added on day 0. CD276^+^ cells were depleted from MLC on day 4 as described above. Purity: CD276^−^ T-cell pools: >99.5%.

### ADCC/CDC activity assay in vitro

CD4^+^ T cells were isolated from PBMCs using Dynabeads™ CD4 Positive Isolation Kit (Thermo Fischer scientific) and incubated with anti-CD276-depleting mAb in the presence or absence of autologous NK cells/autologous plasma for 24 h at 37 °C. Cells were harvested and apoptotic CD4^+^ T cells were analyzed by staining annexin V and 7-AAD according to the manufacture’s instruction.

### In vivo depletion of CD276^+^ cells by a mAb

NSG-Ab°DR4 mice received a single injection in the tail vein with an HLA-DR4^negative^ donor’s CD45RA-depleted memory CD4^+^ T cells (4 × 10^6^ cells/mouse). Autologous NK cells (1 × 10^5^ cells/mouse) were administered once weekly together with CD276-depleting mAb (generated by H-JB) or isotype control twice weekly.

### Lymphocyte isolation from liver, colon, lung, and skin for flow cytometric analysis

Isolation of lymphocytes was conducted as previously described [20].

### Histology analysis

Tissues from NSG-Ab°DR4 mice were fixed in 10% buffered formalin or embedded in paraffin, were HE stained, and were analyzed with mouse anti-human CD3 (Dako). Slides of lungs and livers were assessed by using a semiquantitative scoring system for abnormalities as described [21].

### TCR Vα-repertoire spectratyping

TCR Vα-repertoire complexity was analyzed by CDR3 size spectratyping as published previously [22].

### Identification of the epitope-binding region of TCRs from organ-infiltrating T cells

Single peaks in TCR Vα-repertoire spectratype analysis were subjected to direct sequencing approaches for identification of CDR3 amino acid sequences using BigDye^®^ Terminator v3.1 Cycle Sequencing Kit (Life Technologies, Germany). Sequences were read in an ABI3130 Genetic Analyzer and matched with IMGT, NCBI Blast, and Emboss databases.

### Statistics

Data were analyzed with GraphPad Prism 7.0 to carry out one-way ANOVA followed by Tukey’s multiple comparison test or unpaired *t* tests between groups. For comparison of recipient survival rate, the log-rank test was used to determine statistical significance. *P* < 0.05 was considered statistically significant. Number of repetitions, descriptive measure, and measure of variation depicted are reported in the respective figure legends.

## Results

### CD45RA depletion attenuates alloreactivity in the CD8^+^ T-cell compartment while maintaining alloresponse in CD4^+^ T cells in haplo-MLC

To characterize alloreacting memory T cells, we conducted MLCs between CD45RA-depleted or undepleted responder PBMCs and irradiated haploidentical stimulator PBMCs. Autologous PBMCs used as stimulator cells served as a negative control. T_H_1 cytokines such as IFN-γ and TNF-α, activation markers such as CD69, CD25, and HLA-DR, a proliferation marker Ki-67, and costimulatory/coinhibitory molecules such as ICOS, CD137, CD26, CD276, PD-1, LAG-3, and TIM-3 expressed on lymphocytes were analyzed on day 7 in parallel with surface CD45RA, CCR7, CD28, and CD95 to allocate the cells into memory T-cell compartments [23]. We confirmed that CD45RA depletion downregulated alloreactive IFN-γ^+^ and TNF-α^+^ T cells in CD3^+^ T cells (Supplementary Fig. 1A). Surprisingly, we found that while CD45RA depletion downregulated activation as well as the proliferative status and the secretion of T_H_1 cytokines in CD8^+^ T cells, it did not decrease the secretion of T_H_1 cytokines (Supplementary Fig. 1A) but rather upregulated the activation and proliferative status in CD4^+^ T cells (Supplementary Fig. 2). These findings correspond with a previous report that in clinical settings alloreactivity after CD45RA depletion is mainly mediated by memory CD4^+^ T cells [2]. This phenomenon might be explained by less stringent rules of peptide binding to HLA-class-II molecules and T-cell receptor recognition by CD4^+^ T cells, resulting in more promiscuous (allo)-reactivity in the CD4^+^ subset [24]. For these reasons, our investigation focused on alloreactive memory CD4^+^ T cells hereafter. The expression of coinhibitory molecules, PD-1, and LAG-3 was upregulated in both CD4^+^ and CD8^+^ T cells after CD45RA depletion. CD276 was predominantly expressed on CD4^+^ T cells in MLC, and CD45RA depletion upregulated its expression in CD4^+^ T cells, while on the other hand, its expression in CD8^+^ T cells was not significantly modulated (Supplementary Fig. 2).

### The expression of CD276 correlates with IFN-γ^+^ alloreactive T cells in haplo-MLC

To identify a specific marker for alloreactive CD4^+^ T cells in a CD45RA-depleted graft, we analyzed ten candidate molecules together with IFN-γ and calculated the positive likelihood ratio using sensitivity and specificity to IFN-γ^+^ T cells. We found CD276 expression correlates best with IFN-γ^+^ alloreactive T cells among CD4^+^ T cells but to a lesser degree in CD8^+^ T cells, thus CD276 can be considered a marker for alloreactive CD4^+^ T cells (Supplementary Fig. 1B). The representative scatter plots for CD276 and IFN-γ and for the other markers evaluated and IFN-γ among CD4^+^ T cells are shown in Supplementary Fig. 3. Supplementarily, memory phenotype of CD276^+^CD4^+^ T cells was almost identical with that of IFN-γ^+^CD4^+^ T cells (Supplementary Fig. 4).

### CD45RA/CD276 depletion alleviates alloreactivity both in CD4^+^ and CD8^+^ T cells in haplo-MLC

**Fig. 1:**
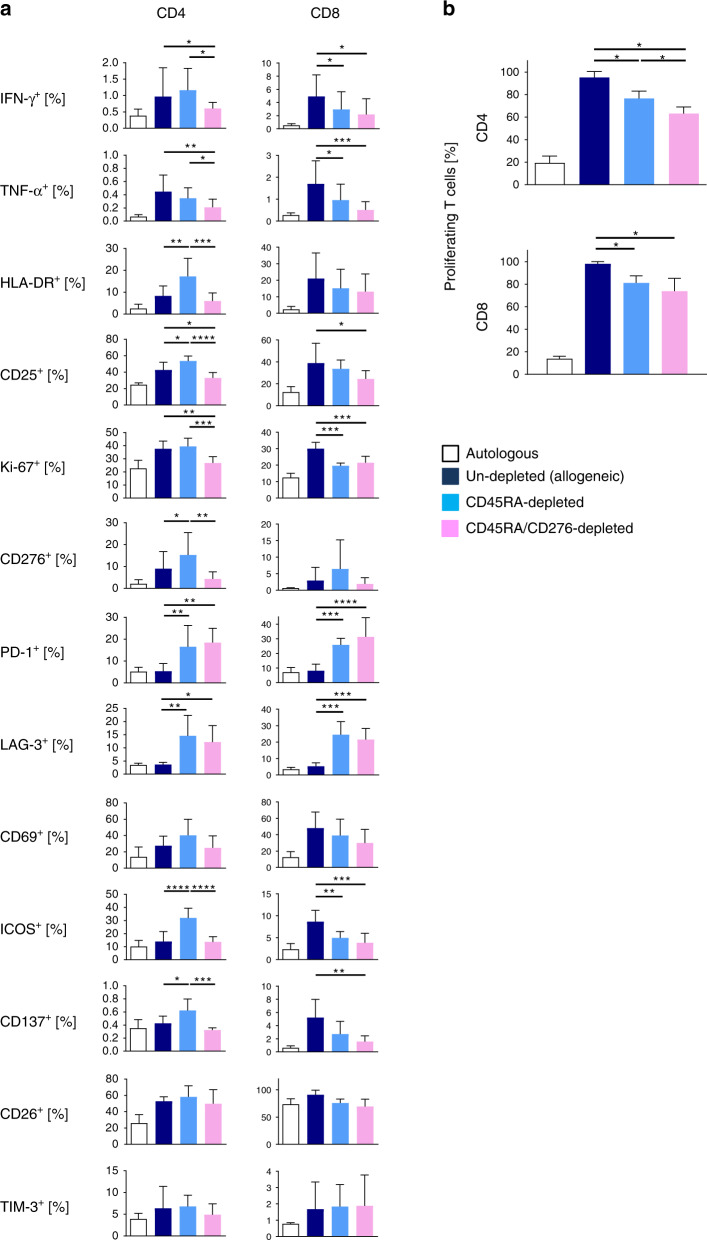
Significant reduction of alloreactive T cells both in the CD4^+^ and CD8^+^ T-cell compartment through CD276 depletion from CD45RA-depleted responder cells in haplo-MLC. After stimulation with irradiated haploidentical PBMCs, CD276^+^ T cells were removed on day 4, and remaining responder cells were tested by flow cytometry. **a** Alloreactive marker expression among CD4^+^ T cells and CD8^+^ T cells at day 11 (*n* = 5). **b** After depletion of CD276^+^ cells from CD45RA-depleted MLCs at day 4, remaining responder cells were CFSE labeled and analyzed in haplo-MLC using irradiated haploidentical stimulator cells or autologous cells for control. Proliferation was measured at day 8 (*n* = 3). All experiments were repeated twice by using the identical donors with consistent results. *P < 0.05, **P < 0.01, ***P < 0.001, ****P < 0.0001 by one-way ANOVA followed by Tukey’s multiple comparison test.

Next, we evaluated CD276^+^ lymphocyte depletion on day 4 from CD45RA-depleted haplo-MLC. Combined depletion of CD45RA^+^ and CD276^+^ cells significantly suppressed activation, proliferation, and secretion of T_H_1 cytokines of CD8^+^ and CD4^+^ T cells at day 11 (Fig. 1a). PD-1 or LAG-3 expression was similar between CD276^+^ and CD276^−^ cells, resulting in spared PD-1 and LAG-3 expression after CD276 depletion.

### CD45RA/CD276-depleted MLCs reduce proliferating T cells compared to CD45RA-sole-depleted MLCs

Besides T_H_1 cytokine-response we examined proliferation of responder PBMCs. These were CFSE labeled day 4 and examined on day 8 of MLC. CD45RA depletion decreased the proliferation of CD4^+^ and CD8^+^ T cells in haplo-MLC quantitatively (CD4^+^ T cells 19.6%; CD8^+^ T cells 17.2%) (Fig. 1b). CD45RA/CD276 depletion reduced the proliferation of CD4^+^ T cells further (17.5%).

### CD45RA/ CD276-depleted grafts maintain reactivity toward common viral antigens

**Fig. 2:**
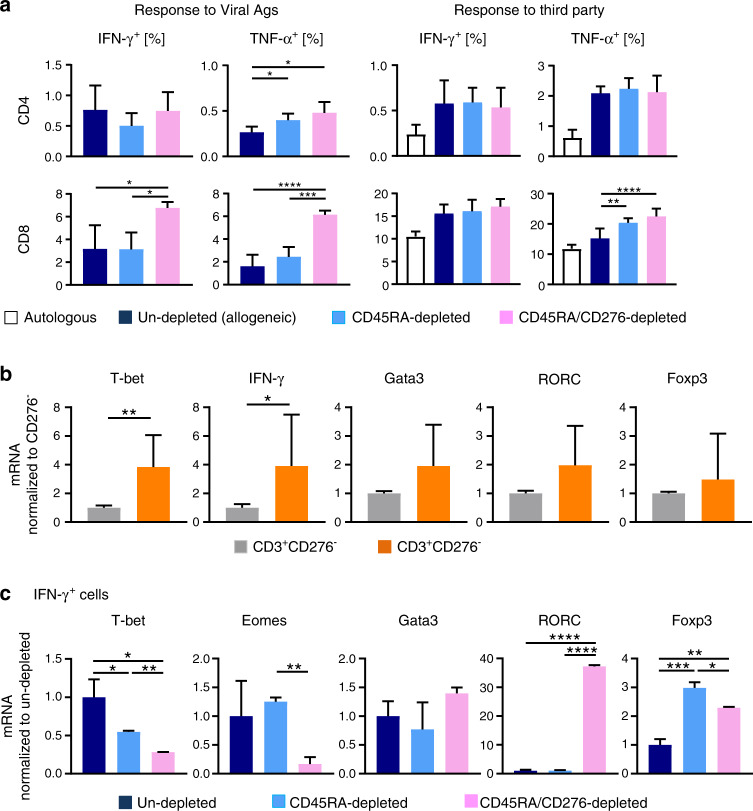
CD45RA/CD276 depletion maintains immune reactivity toward third party and common viral antigens. Remaining alloreactive memory T cells are of T_H_17 phenotype. **a** CD45RA/CD276 depletion maintains immune response to viral antigens. CD45RA/CD276-depleted, CD45RA-depleted, and undepleted donor PBMCs were exposed for 4 h to a virus peptides mix covering the complete sequence of CMV pp65, EBV consensus, and AdV5 antigens and the frequencies of IFN-γ^+^ and TNF-α^+^ T cells were determined (*n* = 4). A viral specific response was calculated as follows: (frequencies of IFN-γ- and TNF-α-positive T cells in culture pulsed with viral peptides mix) − (frequencies of IFN-γ- and TNF-α-positive T cells in culture without peptide mix). CD45RA/CD276 depletion maintains immune response to third party. Following CD276 depletion on day 4, remaining cells were stimulated with third party PBMCs and frequencies of IFN-γ^+^ and CD69^+^ T cells were analyzed at day 11 of haplo-MLC by flow cytometry. CD69 coanalysis served as indicator for activation (*n* = 4). **b** Transcription factor profile of CD3^+^CD276^+^ and CD3^+^CD276^−^ T cells in CD45RA-depleted haplo-MLC at day 4 was analyzed by qPCR (*n* = 4). **c** Transcription factor profile of IFN-γ^+^ T cells from day 11 of CD45RA-depleted haplo-MLC was analyzed by qPCR (*n* = 2). All experiments were repeated at least twice by using the identical donors with consistent results. *P < 0.05, **P < 0.01, ***P < 0.001, ****P < 0.0001 by one-way ANOVA followed by Tukey’s multiple comparison test (**a**, **c**) or unpaired *t* tests (**b**).

To determine whether CD45RA/CD276 depletion maintains antiviral activity in the grafts, we incubated CD45RA/CD276-depleted, CD45RA-depleted, and undepleted donor PBMCs with common viral antigens (EBV, CMV pp65, and AdV5 antigen peptide mix). The frequencies of IFN-γ^+^ and TNF-α^+^ T cells indicate that reactivity to viral antigens persisted in CD45RA-depleted and CD45RA/CD276-depleted memory T-cell subset. CD45RA/CD276 depletion rather enhanced IFN-γ positivity in CD8^+^ T cells and TNF-α positivity in CD4^+^ and CD8^+^ T cells, compared with nondepleted grafts (Fig. 2a).

### CD45RA/CD276 depletion preserves the alloreactivity toward third party PBMCs

To assess whether CD45RA/CD276 depletion maintains third party immune reactivity, we started a second MLC on day 4 with third party PBMCs and examined frequencies of IFN-γ^+^ and CD69^+^ T cells on day 11. CD45RA/CD276 depletion did not reduce responsiveness toward third party, unexpectedly IFN-γ^+^ cells were rather elevated in CD4^+^ or CD8^+^ T cells as was CD69 in CD8^+^ cells, compared with nondepleted grafts (Fig. 2a).

### CD276^+^ T cells are mostly CD4^+^ T cells and of T_H_1 phenotype

CD276^+^ T cells, depleted from the graft from haplo-MLC, were mostly CD4^+^ (95.3 ± 0.9% were CD4^+^CD8, Supplementary Fig. 5). Comparative analysis on day 4 of haplo-MLC showed significantly higher T-bet and IFN-γ mRNA expression in CD276^+^ T cells than in CD276^negative^ counterparts (Fig. 2b) identifying CD276^+^ T cells in haplo-MLC as T_H_1 cells.

### Alloreactive memory T cells after CD45RA/CD276 depletion in vitro are T_H_17 cells

Remaining alloreactive T cells after CD45RA/CD276-depletion at day 4 of haplo-MLC were analyzed by FACS on day 11 of MLC by isolating IFN-g^+^ T cells (purity: IFN-γ^+^ > 96%). RORC^+^ T cells are markedly increased by CD45RA/CD276 depletion, while numbers of T-bet^+^ and Eomes^+^ cells were reduced to 28.3% and 16.8% of control, respectively (Fig. 2c). CD45RA depletion upregulated the expression of Foxp3 in the remaining T-cell pool and this effect was slightly counter-regulated by additional CD276 depletion. The prominent expression of RORC of alloreacting T-cell fraction is biased toward a T_H_17 phenotype.

### CD45RA/CD276 depletion improves GVHD in NSG-Ab°DR4 mice receiving HLA-DR4^negative^ human CD4^+^ T-cell grafts

**Fig. 3:**
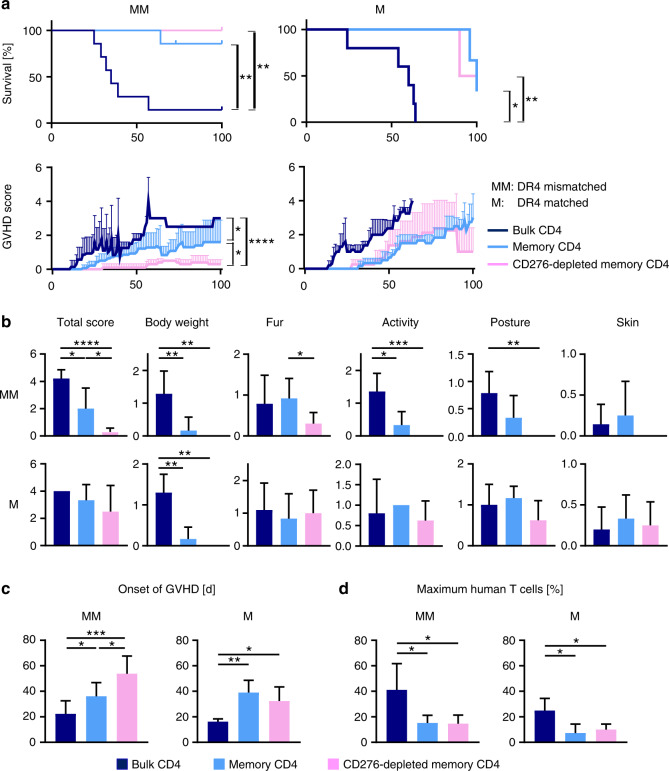
CD45RA/CD276 depletion significantly delayed the onset of GVHD symptoms and remarkably alleviated their severity, leading to the rescue of NSG-Ab°DR4 mice from GVHD mortality in HLA-DR-mismatched setting. NSG-Ab°DR4 mice received one of the three different grafts: HLA-DR4^negative^ or HLA-DR4^positive^ donor’s bulk CD4^+^ T cells (black), or CD45RA-depleted memory CD4^+^ T cells (blue), or CD45RA/CD276-depleted memory CD4^+^ T cells (pink). Mice were scored daily individually for signs of GVHD. **a** Survival, mean + SD of total GVHD scores in each cohort, **b** total scores or respective symptom score before euthanization, **c** schedule of onset of GVHD (days), and **d** the maximum frequency of human T cells among whole PBMCs. (*n* = 6 (bulk CD4^+^ T cells), *n* = 5 (memory CD4^+^ T cells, CD276-depleted memory CD4^+^ T cells)). One mouse in the cohort of memory CD4^+^ T-cell transplantation was excluded from analysis due to sacrification for non-GVHD related complication (uterus prolapse). Shown are the representative data. We performed preliminary experiments at least twice by using the identical donors with consistent results. The Kaplan–Meier method was used to assess survival rate using the log-rank test (**a**). One-way ANOVA followed by Tukey’s multiple comparison test was used (**b**, **c**). *P < 0.05, **P < 0.01, ***P < 0.001, ****P < 0.0001.

Previous clinical data [2] and our in vitro findings suggest recruitment of alloreactive T cells from the CD4^+^ memory compartment. To examine CD45RA/CD276 depletion for the generation of grafts with lower GVHD risk, we used NSG-Ab°DR4 mice as an in vivo model for a human CD4^+^ T-cell-mediated GVHD. This recently developed NSG mouse lacks murine MHC class II but expresses human HLA-DRB1-0401 [25]. Injection of purified human DR4^negative^ CD4^+^ T cells into this mouse was reported to develop a human allo-GVHD originating from mismatched HLA in the absence of xenogeneic GVHD (xeno-GVHD) [25]. NSG-Ab°DR4 mice received one of the three different grafts generated from each of the HLA-DR4^negative^ or HLA-DR4^positive^ donors: (A) bulk CD4^+^ T cells, or (B) CD45RA-depleted memory CD4^+^ T cells, or (C) CD45RA/CD276-depleted memory CD4^+^ T cells. We obtained CD276-deleted memory T cells by depleting CD276^+^ cells on day 4 of MLC between human memory CD4^+^ T cells and mouse PBMCs (Supplementary Fig. 6). Consequently, in both HLA-DR4-mismatched and -matched settings, memory or CD276-depleted memory CD4^+^ T-cell grafts significantly improved survival (Fig. 3a) and extended lifespan of the recipients to the end of the predetermined endpoint of the study, compared to bulk CD4^+^ T-cell recipients (median survival time (MST) = 35 days in case of HLA-DR4^negative^ donors, MST = 60 days in case of HLA-DR4^positive^ donors), and improved clinical GVHD scores (Fig. 3b). In HLA-DR4-mismatched setting CD45RA/CD276 depletion attenuated GVHD and delayed the onset of GVHD further compared to sole CD45RA depletion (Fig. 3c). Contrastively, no significant difference was observed in clinical GVHD scores of CD45RA-depleted and CD45RA/CD276-depleted graft recipients in the HLA-DR4-matched setting (Fig. 3b, c). Images of different individuals per cohort are shown in Supplementary Fig. 7. HLA-DR4-mismatch, induced mild fur symptom in all CD276-depleted memory CD4 T-cell recipients—very discrete loss of some of its glow—without any other signs of GVHD and this symptom was even transient in two out of five mice.

### CD45RA/CD276 depletion weakens the expansion of engrafted human CD4^+^ T cells in the periphery

Mouse peripheral blood was analyzed weekly for the engraftment of human CD4^+^ T cells. CD45RA depletion and CD45RA/CD276 depletion decreased the maximum frequency of engrafted human T cells and extended the time until engraftment compared with nondepleted grafts both in HLA-DR4-mismatched/matched settings (Fig. 3d and Supplementary Fig. 8). Chronological changes of the frequency of human T cells in the peripheral blood and clinical GVHD scores of individual mice are shown in Supplementary Fig. 9.

### CD45RA/CD276 depletion reduces IFN-γ^+^/TNF-α^+^ secretion and activation status of peripheral T cells in DR4-mismatched transplantation

**Fig. 4:**
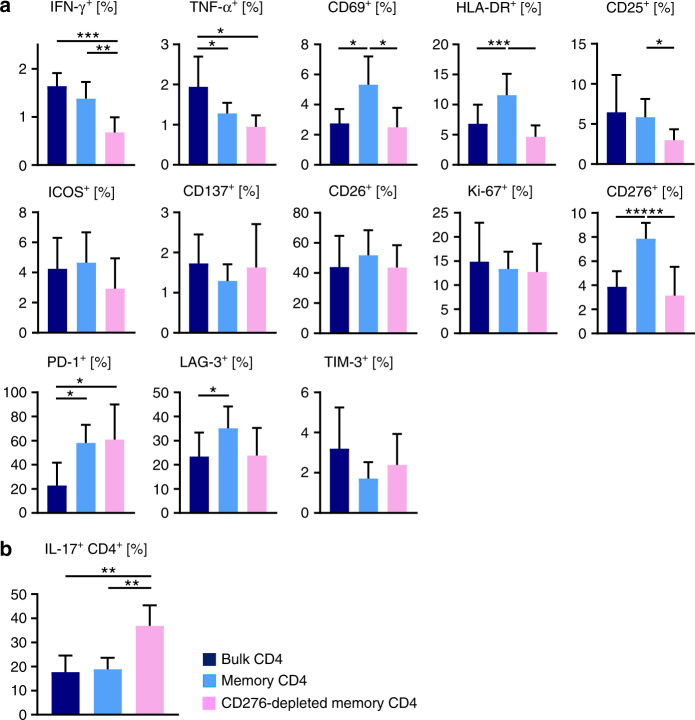
CD45RA/CD276 depletion reduces IFN-γ and TNF-α secretion and activation status of peripheral T cells in DR4-mismatched transplantation. **a** Peripheral blood of DR4 mice was analyzed by flow cytometry for T_H_1 cytokines secretion, activation markers, and costimulatory/coinhibitory molecules when engraftment of human CD4^+^ T cells was achieved (days 12–38). **b** Peripheral IL-17A secreting cells after 4 h stimulation with PMA/Ionomycin were analyzed by flow cytometry when DR4 mice achieved the engraftment of human CD4^+^ T cells. (*n* = 6 (bulk CD4^+^ T cells), *n* = 5 (memory CD4^+^ T cells, CD276-depleted memory CD4^+^ T cells)). Shown are representative data. We performed preliminary experiments at least twice by using the identical donors with consistent results. *P < 0.05, **P < 0.01, ***P < 0.001, ****P < 0.0001 by one-way ANOVA followed by Tukey’s multiple comparison test.

Mouse peripheral blood after DR4-mismatched transplantation was analyzed when human derived T cells first exceeded 5% of whole PBMCs (day 21.4 ± 6.1) yet before GVHD symptoms became evident. Clinical symptoms correlated well with T_H_1-cytokine secretion from engrafted human T cells since CD45RA depletion reduced IFN-γ^+^/TNF-α^+^ CD4^+^ T cells in the periphery, and reduced further by CD45RA/CD276 depletion (Fig. 4a). On the other hand, activation markers such as CD69 and HLA-DR were rather upregulated by CD45RA depletion but downregulated by additional CD276 depletion. Inhibitory co-molecules such as PD-1 and LAG-3 were upregulated by CD45RA and CD45RA/CD276 depletion. Compared to bulk transplantation, CD276 was upregulated on peripheral CD4^+^ T cells in recipients of CD45RA-depleted grafts and was also reexpressed in recipients of CD276-depleted memory CD4^+^ T cells to a comparable level as in recipients of bulk CD4^+^ T-cell grafts (Fig. 4a). Increased numbers of IL-17A-secreting cells were found after CD45RA/CD276 depletion in the remaining T-cell pool (Fig. 4b).

### CD45RA/CD276 depletion markedly attenuates pathological severity of GVHD and reduces infiltration of human T cells in DR4-mismatched transplantation

**Fig. 5:**
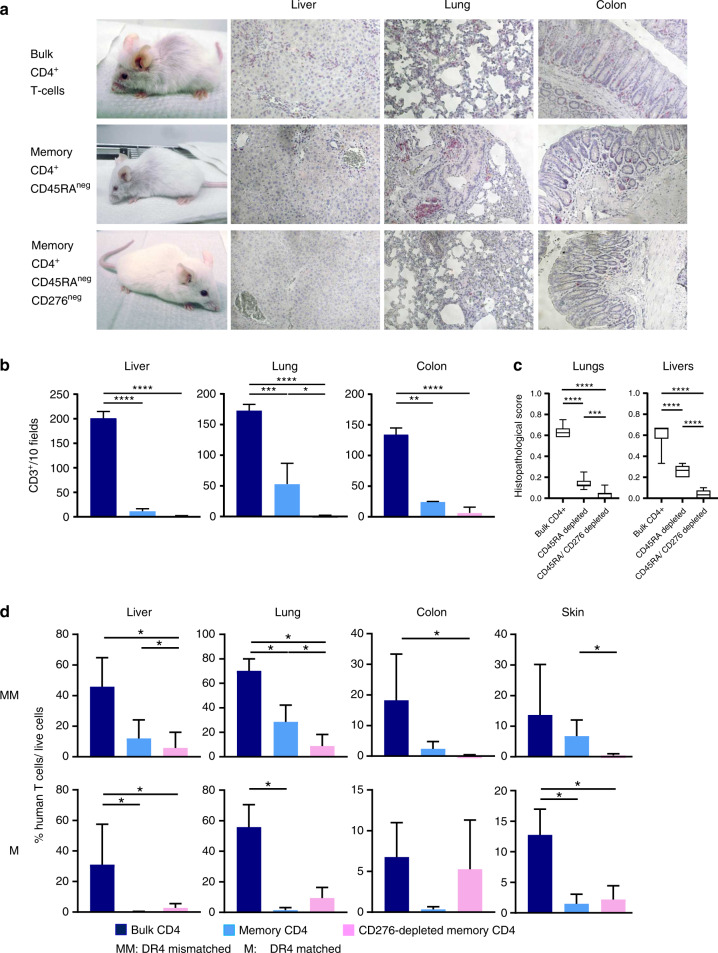
Scarce T-cell infiltration in skin and colon and less infiltration into liver and lung after transplantation of CD45RA/CD276-depleted grafts. **a** Tissues from NSG-Ab°DR4 mice were fixed in 10% buffered formalin and embedded in paraffin for hematoxylin and eosin (HE) staining and immunohistochemical staining with anti-human CD3. **b** CD3^+^ cells were counted in ten representative high-power view fields (×400) under the microscope. **c** Histopathological GVHD score in lungs and liver of individual mice was evaluated in ten representative high-power view fields (×400) under the microscope. **d** Resected tissue specimens were disaggregated into single-cell suspensions, and then stained for flow cytometric analysis. The frequencies of human CD4^+^ T cells were calculated as: %CD4^+^ T cells/total live cells. (*n* = 5 (bulk CD4^+^ T cells), *n* = 4 (memory CD4^+^ T cells, CD276-depleted memory CD4^+^ T cells)). Shown are the representative data. We performed preliminary experiments at least twice by using the identical donors with consistent results. *P < 0.05, **P < 0.01, ***P < 0.001, ****P < 0.0001 by one-way ANOVA followed by Tukey’s multiple comparison test.

The infiltration of human T cells and the tissue damage of GVHD target organs were evaluated when the respective mouse reached endpoint day 100 or endpoint criteria. HE staining and CD3 immunohistochemical staining showed that CD45RA depletion reduced the infiltration of T cells and histological GVHD scores in GVHD target organs and that additional CD276 depletion significantly further decreased lung-infiltrating T cells and histological GVHD scores in DR4-mismatched setting (Fig. 5a–c). In DR4-matched setting, CD45RA depletion reduced the infiltration of T cells and pathological severity in GVHD target organs but there was no significant effect by adding further CD276 depletion (Supplementary Fig. 10).

### CD45RA/CD276 depletion suppresses TNF-α secretion and activation of GVHD target organ-infiltrating T cells in DR4-mismatched transplantation

**Fig. 6:**
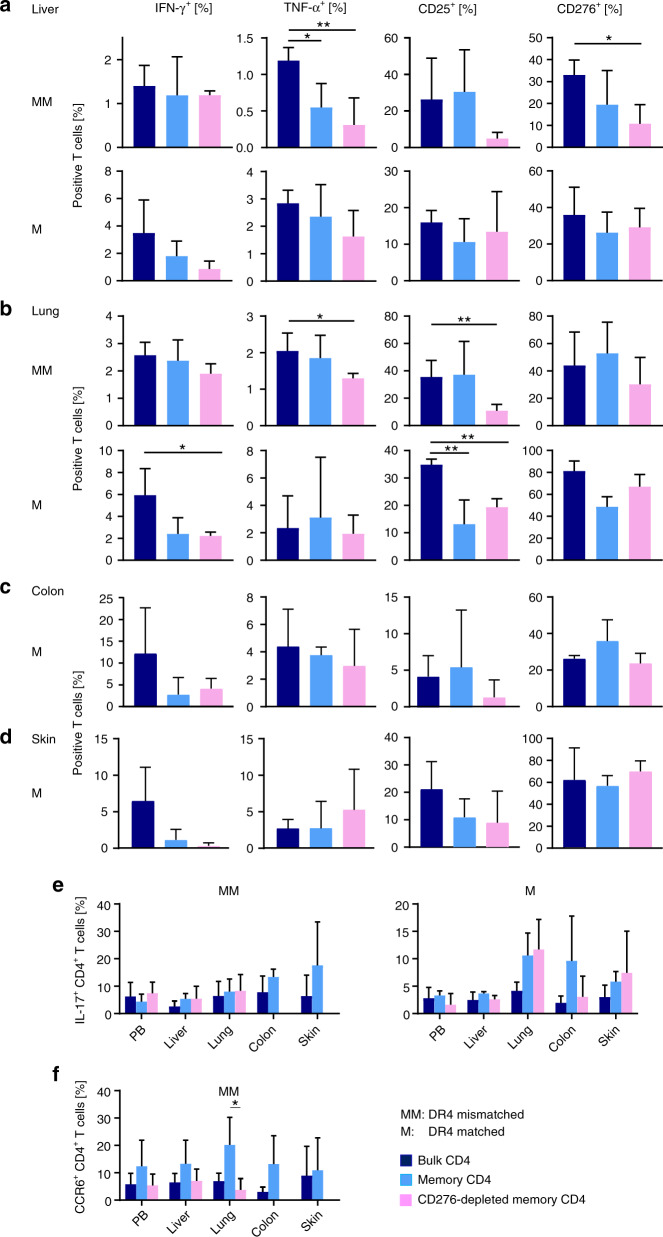
CD45RA/CD276 depletion suppresses TNF-α secretion and activation of GVHD target organ-infiltrating T cells in DR4-mismatched transplantation. **a** Liver-infiltrating human CD4^+^ T cells in DR4-matched and DR4-mismatched transplantation, **b** lung-infiltrating human CD4^+^ T cells in DR4-matched and DR4-mismatched transplantation, **c** colon-infiltrating human CD4^+^ T cells in DR4-matched transplantation, and **d** skin-infiltrating human CD4^+^ T cells in DR4-matched transplantation were analyzed by flow cytometry when mice were sacrificed. **e** Peripheral blood- and organ-derived T lymphocytes were examined by flow cytometry for IL-17A secretion after a 4 h stimulation with PMA/Ionomycin when mice were sacrificed. **f** Peripheral blood- and organ-derived T lymphocytes were examined for CCR6^+^ expression when mice were sacrificed (*n* = 5 (bulk CD4^+^ T cells), *n* = 4 (memory CD4^+^ T cells, CD276-depleted memory CD4^+^ T cells)). Shown are representative data. We performed preliminary experiments at least twice by using the identical donors with consistent results. *P < 0.05, **P < 0.01 by one-way ANOVA followed by Tukey’s multiple comparison test.

We freshly analyzed T cells infiltrating GVHD target organs (skin, liver, colon, and lung) by flow cytometry when the respective mouse reached endpoint or met endpoint criteria. In accordance with pathological findings, significantly less infiltration of liver, lung, colon, and skin was found after transplanting memory T-cell grafts compared with bulk transplants in both DR4-mismatched and -matched settings. Intriguingly, after transplantation of CD276-depleted memory T cells, almost no infiltration was observed in skin or colon and significantly less infiltration in lung and liver compared with sole CD45RA depletion was demonstrated in HLA-DR4-mismatched setting as can be clearly seen in Fig. 5d and Supplementary Fig. 11. In contrast, there was no difference in target organs infiltration between CD45RA depletion and CD45RA/CD276 depletion in HLA-DR4-matched setting (Fig. 5d). Analysis of immunological status of organ-infiltrating T cells revealed that compared with bulk CD4 transplantation, liver-infiltrating T cells exhibited less TNF-α, CD25, and CD276 expression after CD45RA/CD276 depletion and less TNF-α after CD45RA depletion in DR4-mismatched setting (Fig. 6a, further activating markers are shown in Supplementary Fig. 12A). Lung-infiltrating T cells exhibited less TNF-α and CD25 expression in CD45RA/CD276-depleted graft recipients compared to bulk CD4^+^ transplant recipients in DR4-mismatched setting (Fig. 6b, further activating markers are shown in Supplementary Fig. 12B). Thus, CD45RA/CD276 depletion reduces activation status of organ-infiltrating T cells as well as their infiltration rate in target organs in DR4-mismatched setting. By contrast, in DR4-matched setting, there was no significant difference between CD45RA depletion and CD45RA/CD276 depletion in activation status of target organ-infiltrating T cells (Fig. 6a–d and Supplementary Fig. 12A–D). Due to extremely discrete, missing infiltration of skin or colon after CD45RA or CD45RA/CD276 depletion, statistical comparison of skin- or colon-infiltrating T cells was not included. Strikingly, the immunological status of T cells in target organs was quite similar between CD45RA and CD45RA/CD276 depletion. These data suggest that while GVHD target organ T cells are activated to the same extent in these cohorts, absolute number of infiltrated T cells plays a critical role in the severity of GVHD in our model. This finding is consistent with a previous report that infiltration of human T cells correlates with clinical GVHD symptoms in a humanized mouse model [26]. In addition, T_H_17 cells were identified via IL-17A production and CCR6 expression, a chemokine receptor characterizing IL-17-expressing cells [27]. CD45RA/CD276 depletion did not increase recruitment of T_H_17 cells into GVHD target organs in either DR4-matched or mismatched setting (Fig. 6e, f).

### CD45RA/CD276 depletion increases GATA3 and suppressor of cytokine signaling 3 (SOCS3) expression and does not alter RORC expression of GVHD target organ-infiltrating T cells

Pathogenic T_H_17 cells represent highly proinflammatory subpopulation capable of inducing GVHD [28]. Because IL-23 plays a pivotal role discriminating pathogenic Th17 cells [29, 30], we studied the concentration of human IL-23 in the peripheral blood serum when the engraftment of human T cells was achieved. There is no cross-reactivity between human and murine IL-23. IL-23 is mainly produced by antigen presenting cells (APCs), which were not transplanted in our study. The concentration of IL-23 was below the detectable limit by hIL-23 ELISA kit (Thermo Fischer Scientific) in all the mice regardless of the cohorts (data not shown). T-cell activation or differentiation in the spleens was not evaluable since T cells in the spleen were extremely rare and not obtained after CD45RA/CD276-depleted transplantation in DR4-mismatched setting. Since our in vitro data (Fig. 2a) and peripheral blood analysis in NSG-Ab°DR4 mice (Fig. 4b) demonstrated increased T_H_17 compartment after CD45RA/CD276 depletion, to further clarify the contribution of pathogenic T_H_17 cells in this mouse model, we analyzed the expression of RORC, GATA3, T-bet, Foxp3, and SOCS3 in GVHD target organ-infiltrating T cells. T-bet is a key modulator of IL-23-driven pathogenic T_H_17-cell responses [31] and T-bet expression in T_H_17 cells stabilizes pathogenic T_H_17 cells [32, 33]. On the other hand, GATA3 is known as an inhibitor of T_H_17-mediated pathology [34]. RORC/Foxp3-coexpressing cells possess potent suppressive capacity [35]. SOCS3 suppresses T_H_17 differentiation induced by IL-6 and IL-23 [36]. Significant differences of RORC or T-bet expression in any analyzed organ were found neither in DR4-matched nor DR4-mismatched transplant (Supplementary Fig. 13A). CD45RA/CD276 depletion significantly upregulated GATA3 expression in colon and skin in the DR4-mismatched setting and in liver and colon in the DR4-matched setting. The expression level of Foxp3 was generally low and could not be detected by qPCR in most of the samples (data not shown). SOCS3 was upregulated by CD45RA/CD276 depletion in colon and skin in the DR4-mismatched setting and in liver and colon when DR4 was matched. The combination of upregulated GATA and SOCS3 expression with unaltered RORC expression indicates that pathogenic T_H_17 cells were not increased by CD45RA/CD276 depletion in GVHD target organs in this mouse model. In addition, the expression of SOCS3 in donor T cells is reported to negatively regulate GVHD pathophysiology [36], supporting our observation of upregulated SOCS3 expression and amelioration of clinical signs of GVHD by CD45RA/CD276 depletion in DR4-mismatched setting.

### Peripheral CXCR6-expressing T cells are reduced after CD45RA/CD276 depletion in DR4-mismatched transplantation

Since T-cell migration to GVHD target organs is initiated by chemokine gradients produced in these organs, chemotaxis plays a central role in the pathogenesis of GVHD. Therefore, we examined the expression of relevant chemokine receptors on circulating T cells and target organ-infiltrating T cells in DR4-mismatched settings. Although the expression of CXCR6 and CD276 had no remarkable correlation on circulating T cells after memory T-cell transplant (data not shown), CXCR6 expression on circulating T cells was reduced at the time of engraftment in recipients of CD45RA/CD276-depleted grafts (Supplementary Fig. 13B). In contrast, liver, lung, colon, and skin-infiltrating T cells expressed higher CXCR6 than peripheral T cells (Supplementary Fig. 13C), suggesting CXCR6 is involved in the homing/recruitment of T cells to GVHD target organs. Likewise colon-infiltrating T cells express CCR9 higher than the periphery, but no significant numerical difference of CCR9 expressing circulating T cells was observed between cohorts (Supplementary Fig. 14). The expressions of all the other chemokine receptors that we analyzed in this study (CCR3, CCR4, CCR5, and CCR9) were not upregulated in target organs and were not different between cohorts (Supplementary Fig. 14).

### CD45RA/CD276 depletion reduces the diversity of alloreactive T-cell clones in DR4-mismatched transplantation but not in DR4-matched transplantation

To determine the spectrum of TCR diversity of alloreactive T cells, we analyzed the repertoire of 24 different TCR Vα-chains of GVHD target organ-infiltrating T cells. After CD276 depletion, the diversity of the TCR repertoires in CD45RA/CD276-depleted grafts was regular, all of the TCRα chain subfamilies were expressed and showed Gaussian distribution (data not shown). Complexity scores shown in Supplementary Fig. 15A and representative repertoire spectratypes of 5 Va-families shown in Supplementary Fig. 16B indicate a rather limited number of single, clonal expansions than complex polyclonal T-cell pools (Supplementary Fig. 16) after CD45RA/CD276-depleted transplantation than after CD45RA-depleted transplant or bulk transplantation. indicating a rather limited number of single, clonal expansions than complex polyclonal T-cell pools (Supplementary Fig. 16). A biased TCR Vα-chain usage may also suggest recognition of a limited number of target antigens responsible for eliciting GVHD. Therefore, these results suggest that CD276 depletion successfully reduces the number of alloreactive T-cell clones. In contrast, in the DR4-matched setting, the histograms were almost comparably skewed after CD45RA depletion or CD45RA/CD276 depletion (Supplementary Fig. 15A, B).

### Identical T-cell clones occur systemically in DR4-mismatched transplant. Alloreactive T-cell pools contain public TCRs

Single peaks in TCR Vα-repertoire spectratype analysis were subjected to direct sequencing approaches for identification of their epitope-binding CDR3 regions. Amino acid sequences were delineated from these sequences and identified TCRα binding regions (CDR3 regions) of all three cohorts from a representative HLA-DR4^positive^ and HLA-DR4^negative^ donor are given in Supplementary Table 3. We found an identical TCR sequence (TRAV2, TRAJ37, CDR3: CAVEAGNTGKLIFGQG) in skin, liver, and lung in a mouse after CD45RA-depleted transplant and another identical TCR sequence (TRAV3, TRAJ5, CDR3: CAVRDGNTGRRALTFGSG) in colon and skin in a mouse after CD45RA/CD276-depleted transplant in DR4-mismatched setting. This suggests a systemic anti-DR4 alloreactive response in DR4-mismatched setting and is in line with that DR4 is expressed in all organs. On the other hand, no identical TCR sequence was detected in different organs in a mouse in DR4-matched setting, where all minor antigens are of mouse origin thus mismatched and show an organ-specific expression pattern. An identical T-cell clone (TRAV9-2, TRAJ12, CDR3: CIRGLSVCVMGSKGLIFGSG) was identified in lung in the recipient of both memory T-cell transplant and CD276-depleted memory T-cell transplant in the DR4-matched setting, indicating that this alloreactive memory T-cell clone was not eliminated by CD276 depletion using MLC. Public TCRs are identical TCRs or CDR3 regions which can be found in different individuals and have been described in immune responses to viruses, cancer, autoimmune diseases, and alloreactivity [37]. Detected public TCRs toward common virus antigens after CD45RA/CD276 depletion support our in vitro observation that CD45RA/CD276 depletion preserves antiviral activities (Supplementary Table 3). Germline TCRs are defined by recombination of V(D)J gene segments without addition of N-nucleotides and are reported for enhanced affinity toward self-antigens [38]. Germline encoded TCRs were identified exclusively after bulk CD4 T-cell transplantation, suggesting that CD45RA depletion eliminates this aggressive type of T cells (Supplementary Table 3).

### In vivo depletion of CD276^+^ cells improves GVHD in NSG-DR4 mice receiving HLA-DR4^negative^ human memory CD4^+^ T-cell grafts

**Fig. 7:**
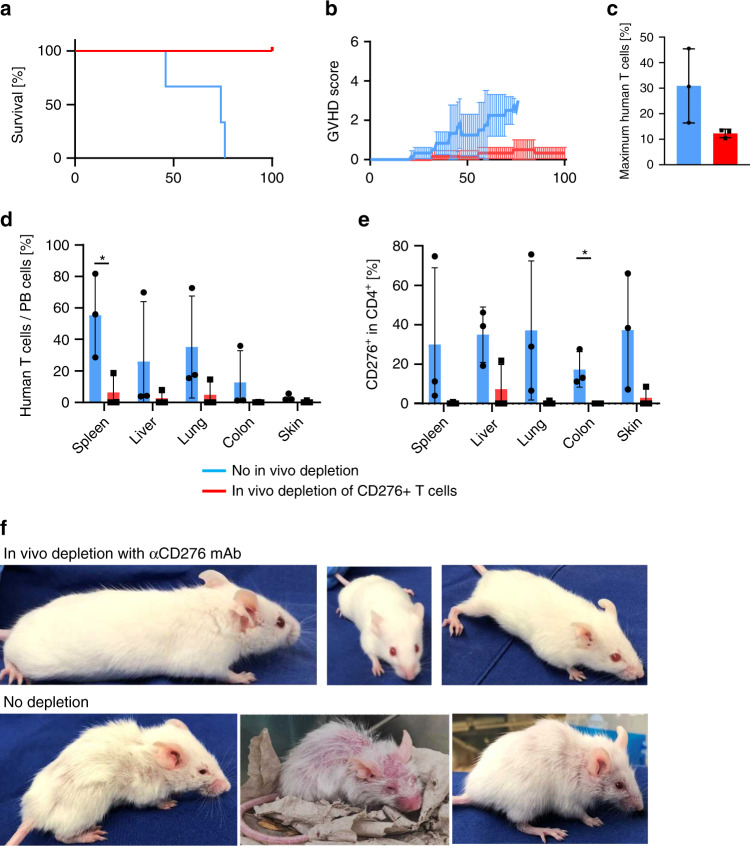
In vivo depletion of CD276 improves GVHD and rescues NSG-Ab°DR4 mice from GVHD mortality. After transplantation of HLA-DR4^negative^ donors’ memory CD4^+^ T cells, NSG-Ab°DR4 mice received αCD276 mAb or isotype control together with identical donors’ NK cells. (*n* = 3). **a** Survival. **b** Mean + SD of total GVHD scores in each cohort. **c** The maximum frequency of human T cells among whole PBMCs. **d** Resected tissue specimens were disaggregated into single-cell suspensions, and stained for flow cytometric analysis. The frequency of human CD4^+^ T cells was calculated as: %CD4^+^ T cells/total live cells. **e** Frequencies of CD276^+^ cells among organ-infiltrating T cells. **f** Images of the individuals from in vivo CD276 depletion (d100) and undepleted cohort (when reaching endpoint criteria) are shown. Shown are representative data. We performed preliminary experiments at least twice by using the identical donors with consistent results. The Kaplan–Meier method was used to assess survival rate using the log-rank test (**a**). Unpaired *t* test was used (**b**–**e**). *P < 0.05, **P < 0.01, ***P < 0.001, ****P < 0.0001.

ADCC activity of the anti-CD276 mAb in the in vivo experiments was congruent with the results of the in vitro experiments (Supplementary Fig. 17). We administered the anti-CD276-depleting antibody together with autologous NK cells to test whether the CD276-depleting mAb attenuates GVHD in this mouse model. Clinical GVHD severity and overall survival were highly significantly improved by the anti-CD276 mAb (Fig. 7a, b). As shown in Fig. 7c, human T-cell expansion had a trend to be weaker after CD276 mAb administration. T-cell infiltration in GVHD target organs and the frequency of CD276^+^ cells in organ-infiltrating T cells were likewise highly significantly reduced (Fig. 7d, e and Supplementary Fig. 18). Images of different individuals shown in Fig. 7f pinpoint the significant difference in transplant outcome. These results strongly suggest mAb-mediated depletion of CD276 T cells for prophylaxis of GVHD in haplo-HSCT. Although weaker human T-cell expansion was observed after CD276 mAb administration, the risk of engraftment failure by CD276 depletion can be considered to be low, since human T cells were indeed engrafted in these mice and the peripheral human T-cell expansion was most likely enhanced by GVHD-mediated systemic inflammation, which was considered to be severer in non-CD276-depleted mice.

## Discussion

Haplo-HSCT using CD45RA-depleted allograft warrants improvement for the prevention of GVHD. More specifically, the nature of alloreactive T cells in the antigen-experienced T-cell compartment remains to be elucidated. The present study, therefore, examined costimulatory/coinhibitory molecules expressed by alloreactive T cells in haplo-MLC and identified CD276 as a marker for alloreactive T_H_1 cells in CD45RA-depleted grafts. Next, we demonstrated in vitro that the depletion of CD276-expressing T cells from CD45RA-depleted alloreacting grafts significantly attenuates the alloresponse to recipient cells, while maintaining and even increasing immune responses toward third party and common viral antigens, respectively. Since NSG mouse-based assessment of GVL effect on human leukemia cells or cell lines by unmanipulated human hematopoietic cells has not been established, we could not demonstrate direct evidence whether CD45RA/CD276 depletion negatively impacts GVL or not. However, sustained and potent third party reactivity in vitro is suggestive for the preservation of GVL activity in these transplants. Finally, we showed in a highly sensitive human allo-GVHD model in vivo that in HLA-mismatched transplantation, CD45RA/CD276-depleted CD4^+^ grafts delay the onset of GVHD and attenuate the severity compared with sole CD45RA-depleted CD4^+^ grafts. CD45RA/CD276 depletion almost completely eliminated skin and colon infiltration by T cells and exhibited only scarce infiltrates with decreased activation status in lung and liver. The alleviated target organ infiltration of donor T cells was partially explained by reduced expression of CXCR6 in the periphery. On the other hand, HLA-matched transplant in the same mouse model resulted in no improvement of clinical GVHD by additional CD276 depletion compared with sole CD45RA depletion. These results suggest that CD276-expressing memory CD4^+^ T cells represent GVHD-triggering T_H_1 cells and their selective depletion from the donor graft can provide better control of GVHD in HLA-mismatched HSCT such as in haplo settings but not in HLA-matched HSCT such as transplant from matched unrelated donor where many if not most minor antigens are mismatched.

B7-H3 (CD276) is a B7 family member whose receptors or function in immune regulation have yet to be clearly defined. CD276 is induced upon activation in T cells, B cells, NK cells, monocytes, and dendritic cells (DCs) [39–42]. The role of CD276 in APCs and immune regulation has been studied intensively. CD276 expressed by DCs binds an as yet unknown counter-receptor (possibly TREML2) [43] on activated T cells, and appears to enhance proliferation of CD4^+^ and CD8^+^ T cells and to selectively increase IFN-γ expression [40]. However, experimental data obtained in several studies indicate complex biological functions of CD276. In one study in CD276-deficient mice, Suh et al. report that CD276 is a negative regulator that preferentially affects T_H_1 responses, as it downregulates T_H_1-mediated immune responses and inhibits the secretion of the cytokine IFN-γ [44]. This finding is in line with the observations from Veenstra et al. who found in a CD276-deficient model [15] that T cells in an allogeneic setting show increased T-cell proliferation, thereby worsening GVHD. Other studies have shown that soluble CD276 is released from monocytes, DCs, and activated T cells and is detectable in normal human serum [45] and that recombinant CD276 protein can induce T-cell proliferation and enhance IFN-γ and IL-10 secretion [46]. To add another layer of complexity, it has also been shown that CD4^+^ T cells upon activation and CD8^+^ T cells constitutively express TREML2, the potential ligand for CD276 [43], and that myeloid cells in inflammatory conditions upregulate TREML2 [47]. This suggests that APCs by expressing TREML2 and CD276 may enhance CD4^+^ and CD8^+^ T-cell activation, proliferation, thereby increasing their IL-2 and IFN-γ production [43]. On the other hand, the characterization of CD276 expressed by T cells, especially regarding the impact of CD276 of donor T cells on GVHD, remains poorly understood. Therefore, in the present study, we compared CD276^+^ T cells with CD276^negative^ T cells and depleted CD276^+^ T cells specifically by exploiting ex vivo MLC, to reveal the physiological role of CD276 expressed on T cells.

T_H_1 cells are the main drivers of aGVHD and high levels of T_H_1-related cytokines such as IFN-γ are found in all target tissues of aGVHD [48]. Our results consistently show that severity of clinical GVHD symptoms correlates with T_H_1 cells. Contrastingly, a conclusive role of T_H_17 in GVHD development has not yet reached a consensus [49–51]. While certain subpopulations of T_H_17 cells are considered to be positive regulators of immune responses by producing proinflammatory cytokines, including IL-17A, IL-17F, and IL-22 (pathogenic T_H_17 cells), other subpopulations of T_H_17 cells can negatively regulate immune responses by secreting immunosuppressive factors, such as IL-10 (nonpathogenic T_H_17 cells) [52]. We found increased numbers of T_H_17 cells in the periphery in CD45RA/CD276^negative^ graft recipients at the time of the engraftment, but T_H_17 cells did not increase in GVHD target organs. In addition, we show there is no evidence of transcription factors indicating increased pathogenic T_H_17 cells. These results indicate that T_H_17 cells are nonpathogenic and not contributive for GVHD in this mouse model. With respect to clinical settings, whether protective or pathogenic Th17 cells are generated is mainly dependent on the presence or absence of phenotype stabilization by IL-23 [30, 53]. IL-23 serves as a pivotal factor that drives inflammatory functions of pathogenic Th17 cells through the induction of alternate lineage-specific transcription factors such as *Tbx21* and effector genes such as *Il22*, *Csf2*, and *Ifng*, while maintaining the core Th17 program such as *Rorc* and *Il17* [29, 30]. Since human IL-23 was not detectable in the serum of NSG-Ab DR4 mice, while T_H_17 cells were nonpathogenic and not expanded in this mouse model due to lack of human IL-23, whether Th17 cells become pathogenic or not in the clinical setting where IL-23 exists remains to be elucidated. Since the incidence of cGVHD after CD45RA-depleted graft transplantation is remarkably low [3], it can be speculated that the T-cell compartment involved in chronic GVHD is already eliminated by CD45RA depletion. Therefore, in this study, we focused on the effect of CD276 depletion additional to CD45RA depletion on acute GVHD.

Chemotaxis plays a central role in the tissue-specific pathogenesis of GVHD. The preferential migration of T cells to gut and liver tissue is mediated by their expression of α4β7/CCR9 or CCR5/CXCR6 and their respective ligands expressed in gut and liver tissues. Accordingly, a phase 2 trial reported that CCR5 blockade prevented GVHD of liver and gut before day 100 [54]. The preferential homing of T cells to lung and skin tissues is mediated by their expression of CCR3, CCR4, and CCR6, whose ligands are present in lung and skin epithelia [20]. In this study, we demonstrate that CXCR6, which is expressed by T cells upon activation and is upregulated in several inflammatory diseases, was downregulated on T cells after CD45RA/CD276 depletion in DR4-mismatched setting. The CXCR6^+^CD4^+^ T-cell subset consists of terminally differentiated effector cells that serve as the major source of effector cytokines in inflamed tissue [55]. Thus, CXCR6^+^ T-cell depletion through CD276 depletion resulted in improved tissue integrity.

TCR-sequencing results of this study suggest that alloreactive T cells systemically infiltrate GVHD target organs in HLA-mismatched transplantation, while systemic infiltration was not confirmed in the HLA-matched transplant situation (Supplementary Table 1). Although some mHAgs are expressed ubiquitously, others possess tissue-restricted properties. Tissue-specific expression of mHAgs induces different patterns of TCR repertoire complexity in different organs. Since organ-specific mHAgs are not expressed by PBMCs, MLC using PBMCs cannot eliminate alloreactive T cells specific for organ-specific antigens. This explains why ex vivo graft engineering, i.e., depleting CD276^+^ cells after donor recipient MLC dramatically decreased the severity of GVHD in an HLA-mismatched setting but did not improve the clinical outcome in an HLA-matched xeno-GVHD model. Therefore, the strategy of depleting CD276^+^ cells from the grafts is considered more suitable for HLA-mismatched HSCT.

When aiming to bring CD276-depleted graft into clinical practice, a stepwise protocol would include: (A) CD34-positive selection, (B) CD45RA depletion from the CD34-negative fraction, (C) stimulation of the CD45RA-depleted fraction with patient PBMCs, and (D) depletion of the CD276^+^ cells from the activated CD45RA-depleted cells and quality testing, before infusion to the patients. The performance of this elaborate graft engineering procedures is a considerable hurdle. On the other hand, immune suppressive agents such as steroids, calcineurin inhibitors, and mycophenolate mofetil theoretically increase the risk of infection and disease relapse/de novo malignancy in addition to the side effects of the drugs themselves. Posttransplantation cyclophosphamide regimen to block alloactivation in vivo needs prophylactic immune suppressive agents and therefore the risk of disease relapse/de novo malignancy should be further evaluated by the long-term follow up. Alpha beta TCR T-cell/B cell-depleted transplantation has 25% probability of developing acute GVHD and eventually needs immunosuppressive therapy [56]. In this respect, if CD45RA/CD276 depletion enables to save immune suppressive agents for control of GVHD, its clinical benefit should be balanced with the hurdle of complex cell processing procedures.

An alternative for this time-consuming and costly procedure with regulatory challenges might be in vivo depletion strategy, as we show in this study that recipients of the same graft have a complete different outcome depending on the administration of a CD276-depleting antibody. These data allow to conclude that CD276 depletion of memory T cells with a potent CD276 antagonistic antibody may be one option to ameliorate acute GVHD after transplantation. Abatacept blocks CD28-mediated T-cell costimulation by specifically binding to CD80/CD86 on APCs. It is already in clinical use for rheumatic arthritis and has yielded promising results to inhibit GVHD in early phase clinical trials [57, 58]. However, CD28-mediated signal drives proliferation, activation, and effector functions of memory CD4^+^ and CD8^+^ T cells [59, 60], therefore CD28 signal blockade by abatacept may lead to ceasing adaptive T-cell responses in total. In this respect, further long-term evaluation is warranted to conclude whether abatacept increases the risk of infection and disease relapse. In contrast, since CD276 is a more specific marker for alloreacting memory CD4^+^ T cells—and alloreactive T cells in CD45RA-depleted grafts are preferentially recruited from the CD4^+^ compartment [2]—in vivo depletion of CD276^+^ T cells may lower the risk of infection and disease relapse by preferentially depleting alloreacting CD4^+^ T cells while sparing virus-specific and cancer cell-specific T cells.

Thus, more studies are needed to investigate the intricate CD276 signaling network to find a strategy how CD276 can be safely and efficiently targeted to prevent and ameliorate GVHD in vivo in clinical settings.

## Supplementary information

Supplementary Figures and Tables
