# Supplementary material for: Removal of CD276+ cells from haploidentical memory T-cell grafts significantly lowers the risk of GVHD
Source: Bone Marrow Transplant. 2021 May 11;56(10):2336–54. doi: 10.1038/s41409-021-01307-9 (PMC8486669; doi:10.1038/s41409-021-01307-9)
Supplement: Supplementary file 1 — Supplementary Figures and Tables [file 41409_2021_1307_MOESM1_ESM.pdf]

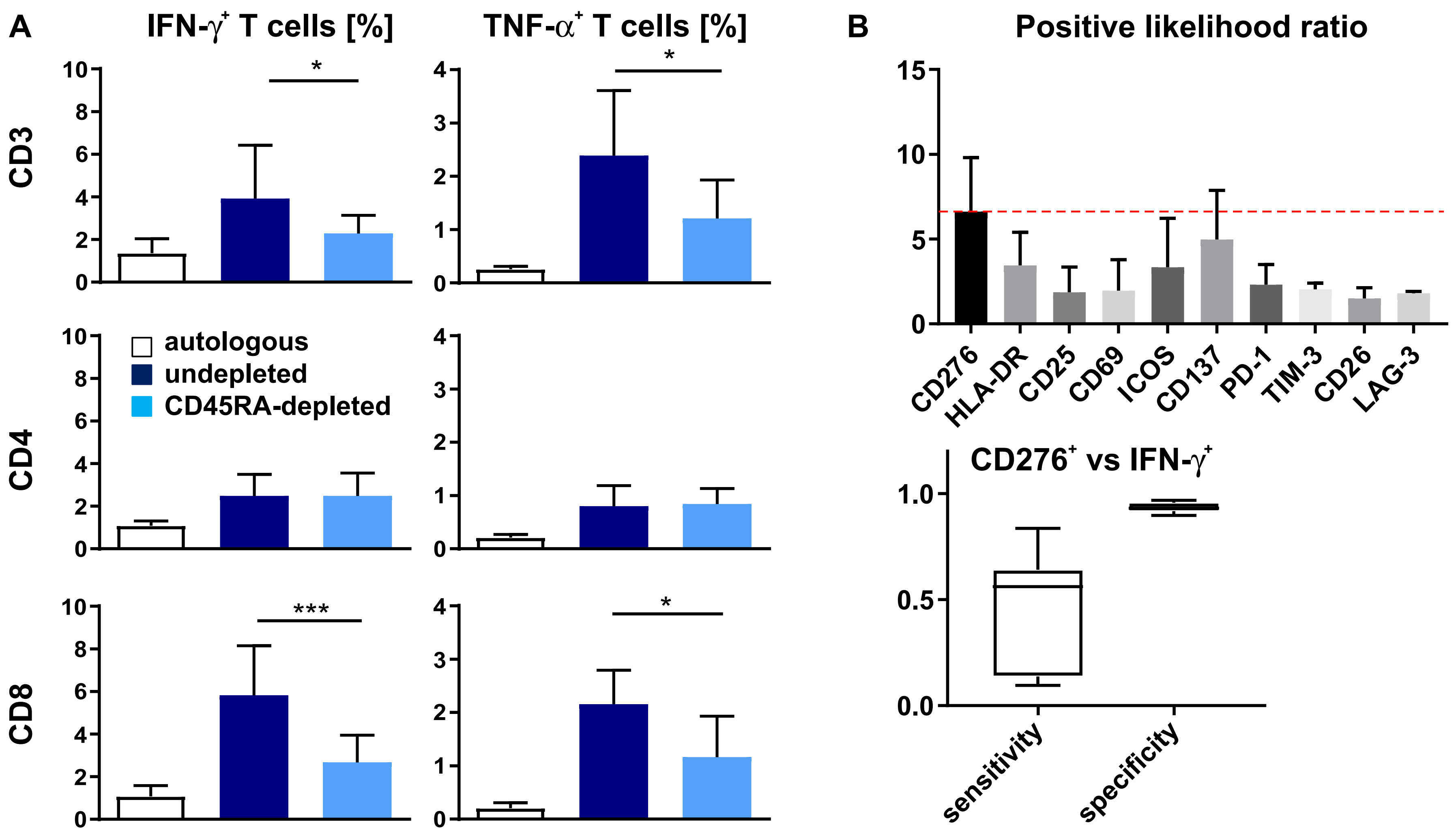

Supplementary Figure 1: CD45RA-depletion attenuates alloreactivity in CD8+ but not in CD4+ T cells. CD45RA-depleted and whole PBMCs were stimulated at a 1:1 ratio with haploidentical irradiated PBMCs and tested for cytokine secretion at day 7. (A) IFN-γ+ and TNF-α+ CD4+ and CD8+ T cells among CD3+ (n=4). (B) Positive likelihood detection of IFN-γ+ CD4+ alloreactive T cells calculated by (sensitivity) / (1- specificity) (n=4). Sensitivity and specificity of CD276+ T cells in relation to IFN-γ+ alloreactive T cells (n=4). Sensitivity for molucle X= X+IFN-γ+ (%)/(X+ IFN-γ+ (%) + X- IFN-γ+(%)), Specificity= X- IFN-γ- (%)/(X- IFN-γ-(%) + X+ IFN-γ- (%)), Positive Likelihood Ratio=Sensitivity/(1-Specificity). *P < 0.05, ***P < 0.001 by one-way ANOVA followed by Tukey's multiple comparison test.

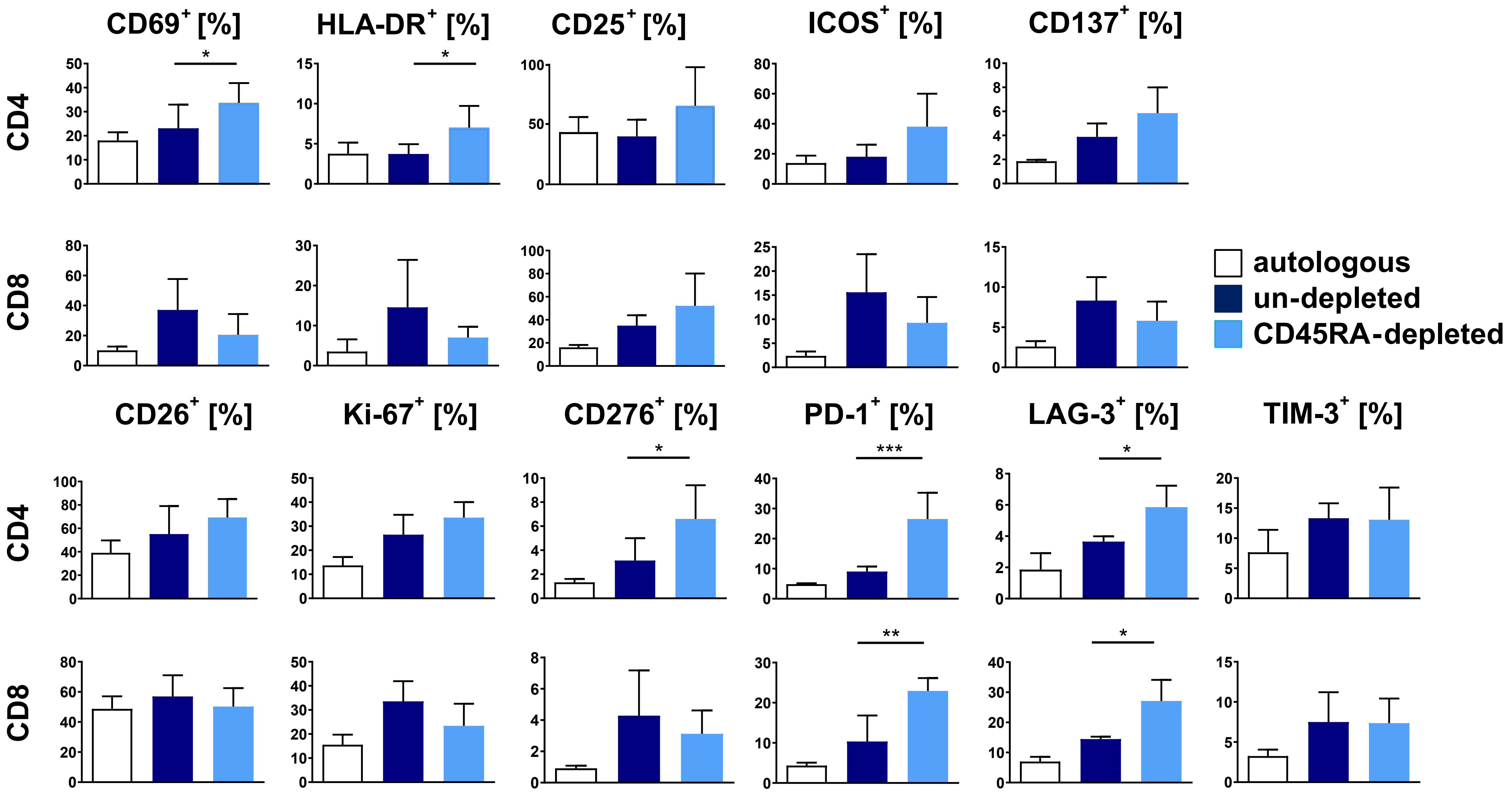

Supplementary Figure 2: CD45RA-depletion attenuates alloreactivity in CD8^{+} but not CD4^{+} T cells. CD45RA-depleted and whole PBMCs were stimulated at a 1:1 ratio with haploidentical, irradiated PBMCs and tested for activation/ proliferation markers and co-receptor expression at day7 (n=4). *P < 0.05, **P < 0.01, ***P < 0.001 by one-way ANOVA followed by Tukey's multiple comparison test.

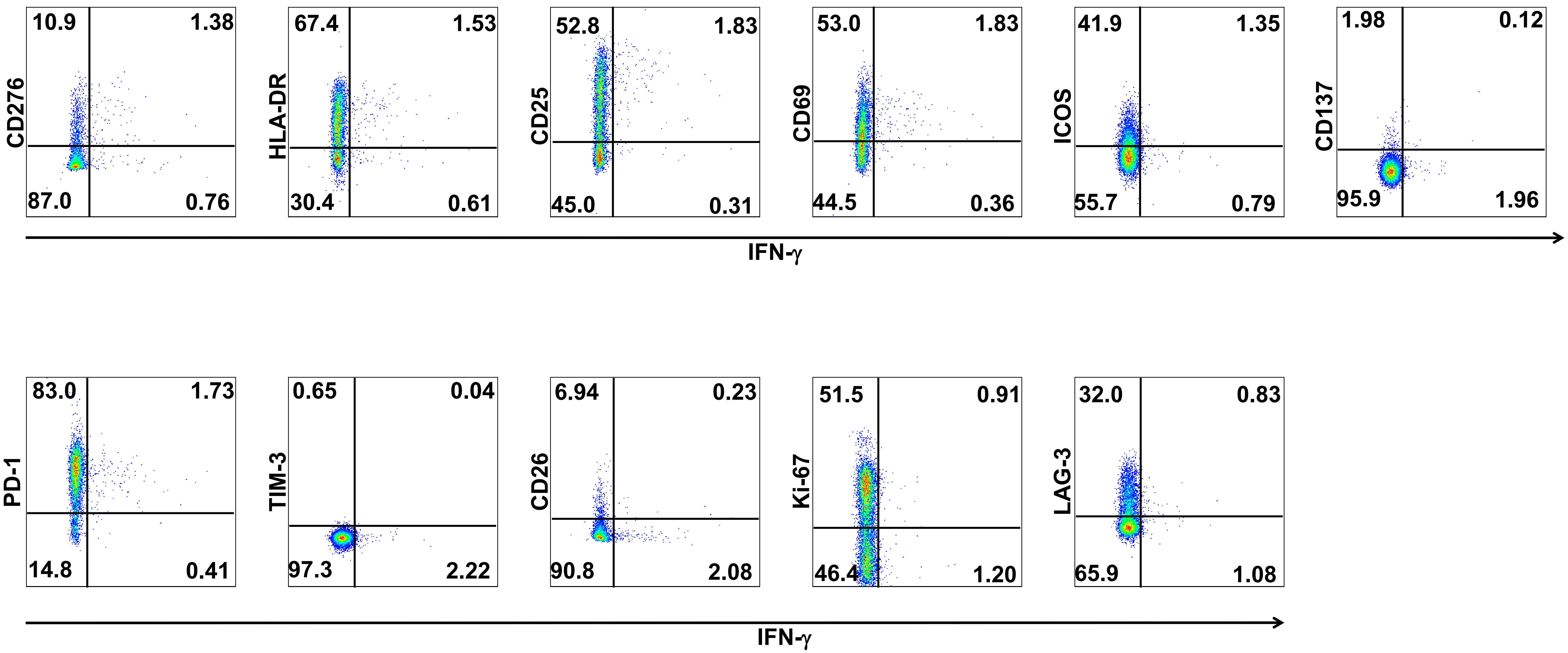

Supplementary Figure 3: Correlation of activation/ proliferation markers and co-receptors with **IFN-γ.**
CD45RA-depleted PBMCs were stimulated at a 1:1 ratio with haploidentical irradiated PBMCs and tested for activation/ proliferation markers and co-receptors expression at day 7.The representative scatter plots for CD276 and IFN, and for the other markers evaluated and IFN among $CD4^{+}$ T cells are shown. The values in scatter plots indicate the percentage of the cells in each quadrant region.

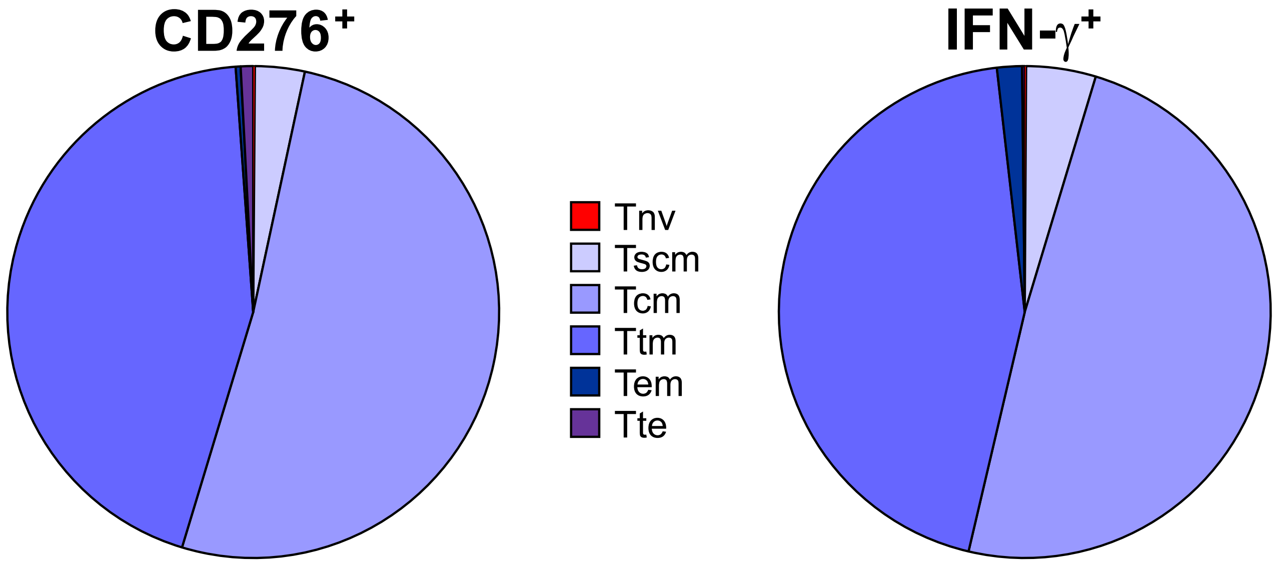

Supplementary Figure 4: Memory phenotype of $CD276^{+}CD4^{+}$ T cells is almost identical with that of IFN-$\gamma^{+}CD4^{+}$ T cells. CD45RA-depleted PBMCs were stimulated at a 1:1 ratio with haploidentical, irradiated PBMCs and immunological memory phenotype of $CD276^{+}CD4^{+}$ T cells and of IFN-$\gamma^{+}CD4^{+}$ T cells was analyzed at day7. Data from one representative donor out of four are shown.

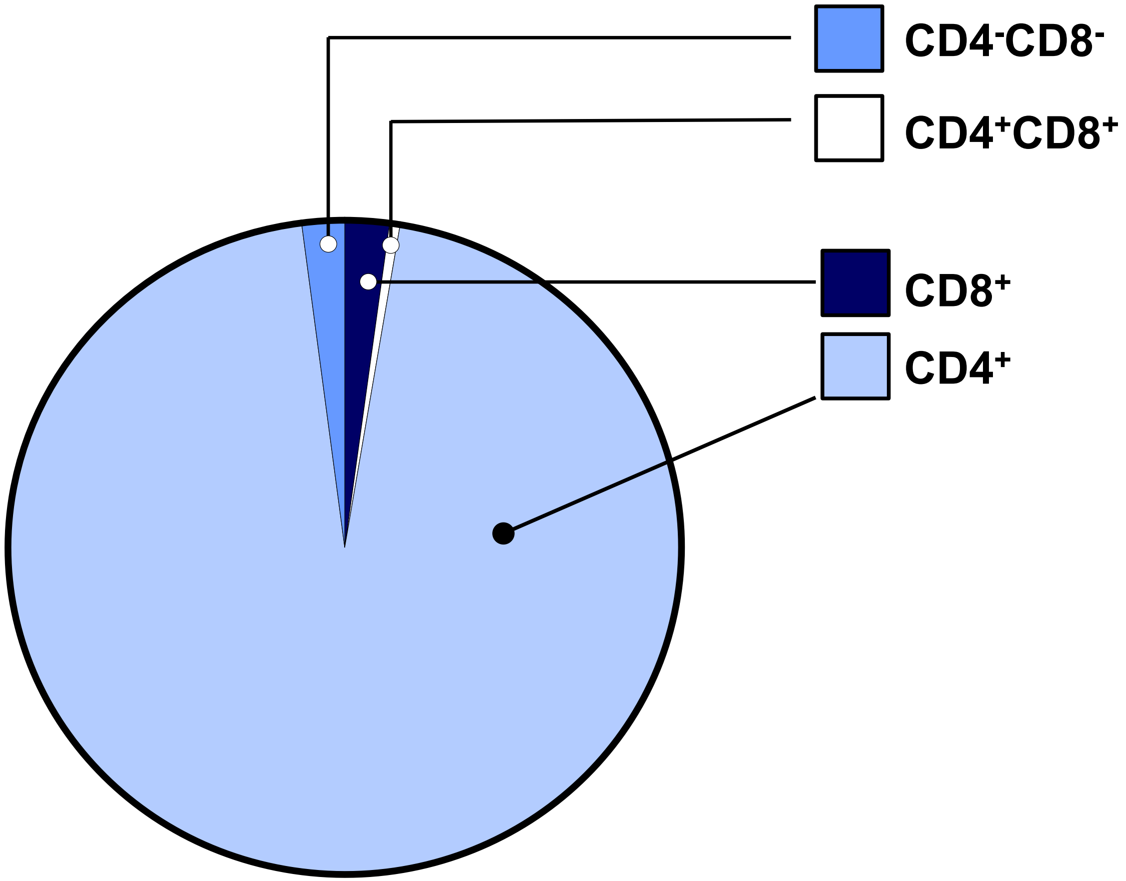

Supplementary Figure 5: Magnetically isolated $CD276^{+}$ T cells from the graft after haplo-MLC, are mostly $CD4^{+}$. CD45RA-depleted PBMCs were stimulated at a 1:1 ratio with haploidentical irradiated PBMCs and $CD276^{+}$ T cells were analyzed for CD4/CD8 positivity at day7 (n=5). Mean values are shown.

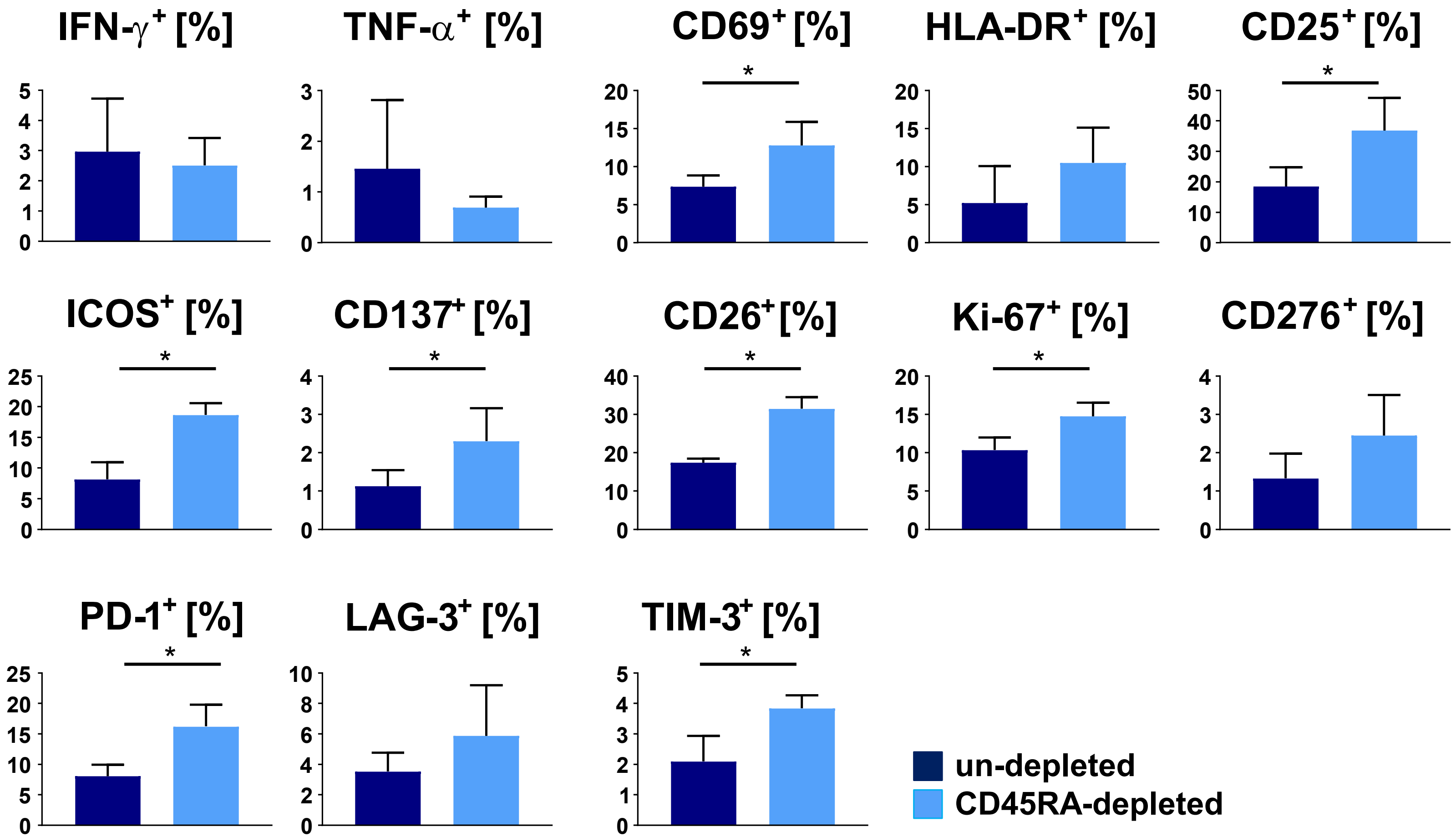

Supplementary Figure 6: CD45RA-depletion does not reduce IFN-γ/TNF-α secretion, but rather up-regulates activation markers such as CD69 and CD25, Ki-67, and co-stimulatory/co-inhibitory molecules such as ICOS, CD137, CD26, CD276, and PD-1 in MLC between $DR4^{negative}$ human $CD4^{+}$ T cells and $DR4^{+}$ mouse PBMCs. $DR4^{negative}$ human bulk or memory $CD4^{+}$ T cells were stimulated at a 3:1 ratio with $DR4^{+}$ mouse PBMCs in MLC (n=3). *$P < 0.05$ by one-way ANOVA followed by Tukey's multiple comparison test.

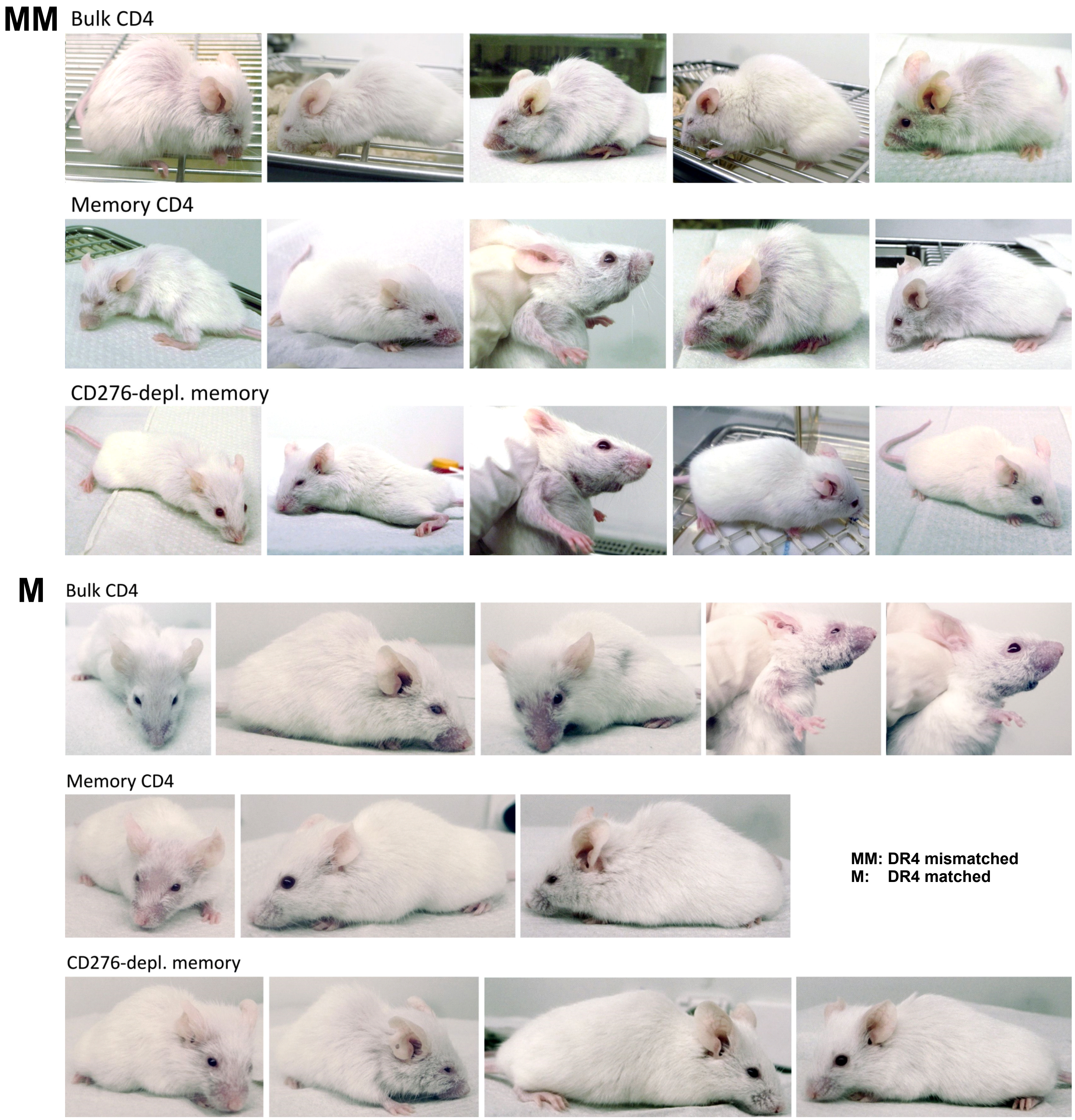

Supplementary Figure 7:

In both HLA-DR4-mismatched and matched settings, memory or CD276-depleted memory $CD4^+$ T cells significantly improves clinical GVHD symptoms. In HLA-DR4-mismatched setting CD45RA/CD276-depletion attenuates GVHD symptoms further compared to sole CD45RA-depletion while no significant difference was observed in the clinical GVHD symptoms of recipients receiving CD45RA-depleted and CD45RA/CD276-depleted grafts in the HLA-DR4-matched setting. Images of different individuals from bulk, memory or CD276-depleted memory cohort respectively in the DR4 mismatched and DR4 matched setting are shown.

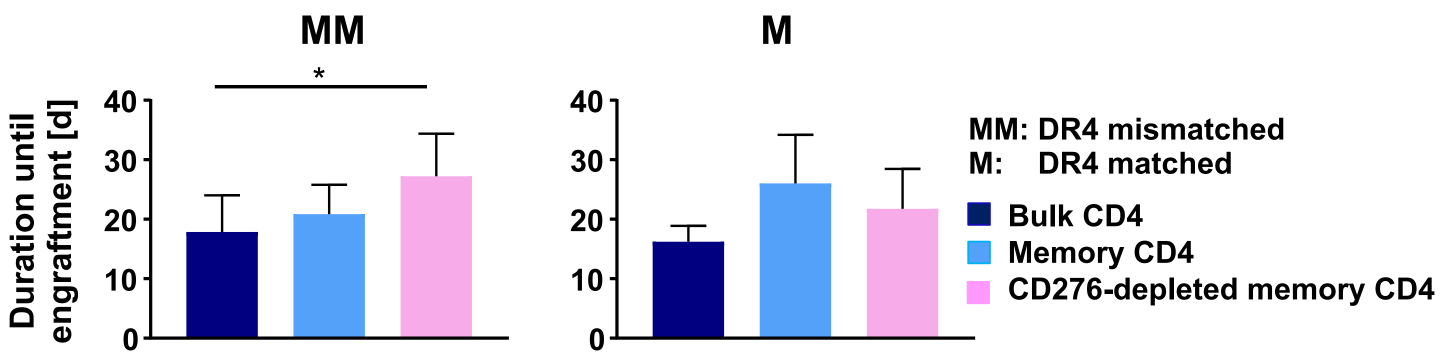

Supplementary Figure 8: CD45RA/CD276-depletion extended the time until engraftment compared with non-depleted grafts in HLA-DR4-mismatched settings. Time periods until first detection of >5% human cells in whole PBMCs (days) are shown. *P < 0.05 by one-way ANOVA followed by Tukey's multiple comparison test.

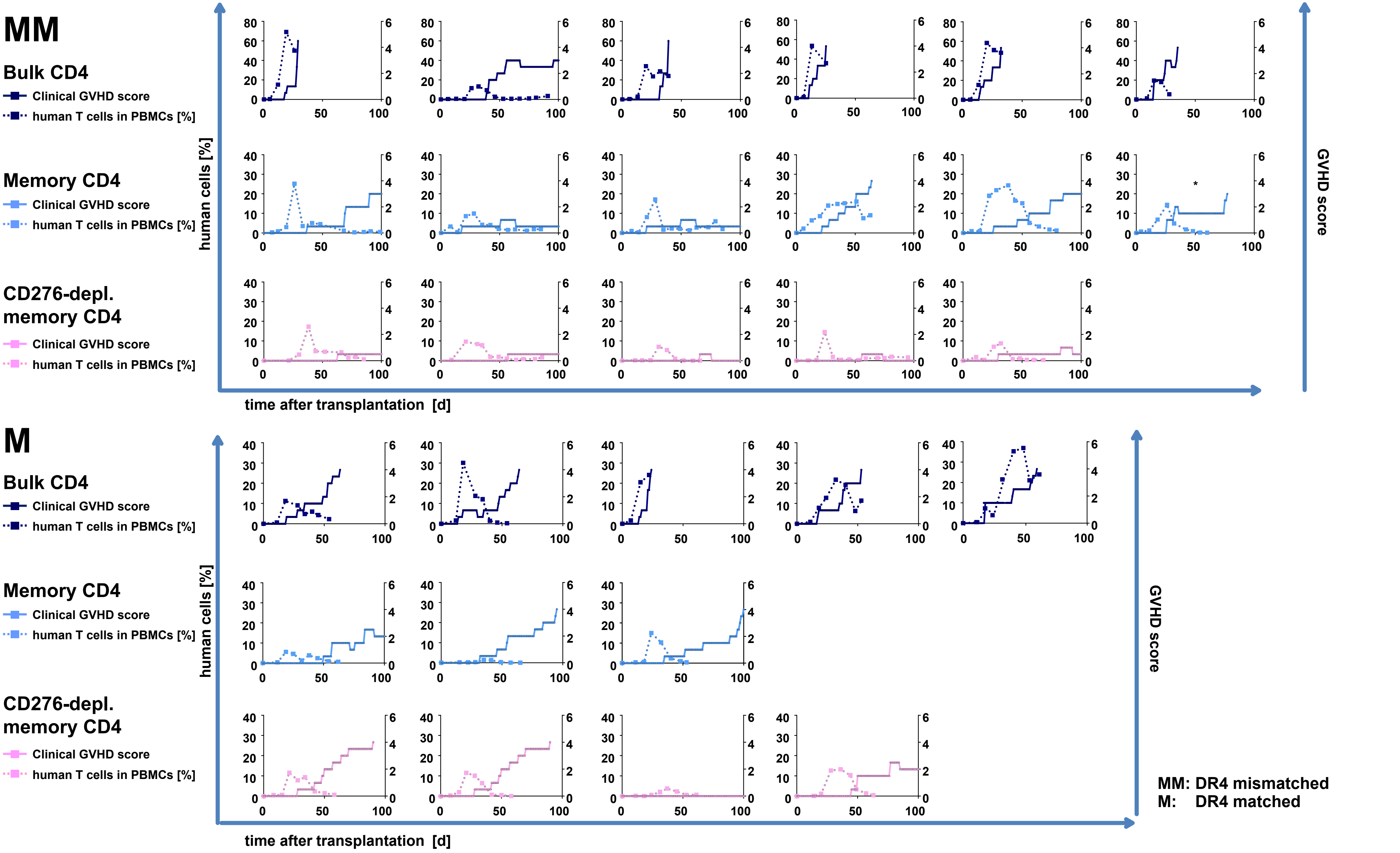

Supplementary Figure 9: Chronological change of the frequency of human T cells in the peripheral blood and clinical GVHD score of individual mice. Individual mice were scored daily for the summation of five clinical parameters of GVHD (posture, activity, fur, skin and weight loss). The frequency of human T cells in the mouse peripheral blood was monitored weekly. Mice were sacrificed when reaching a single score of 1.5 or exceeding clinical score of 4 or when reaching endpoint of the study (day100). One mouse in the cohort of memory $CD4^{+}$ T-cell transplantation from a $DR4^{negative}$ donor (designated by *) was excluded from analysis due to sacrification for non-GVHD related complication (uterine prolapse). Left y-axis: % human T cells in mouse PBMCs. Right y-axis: GVHD score.

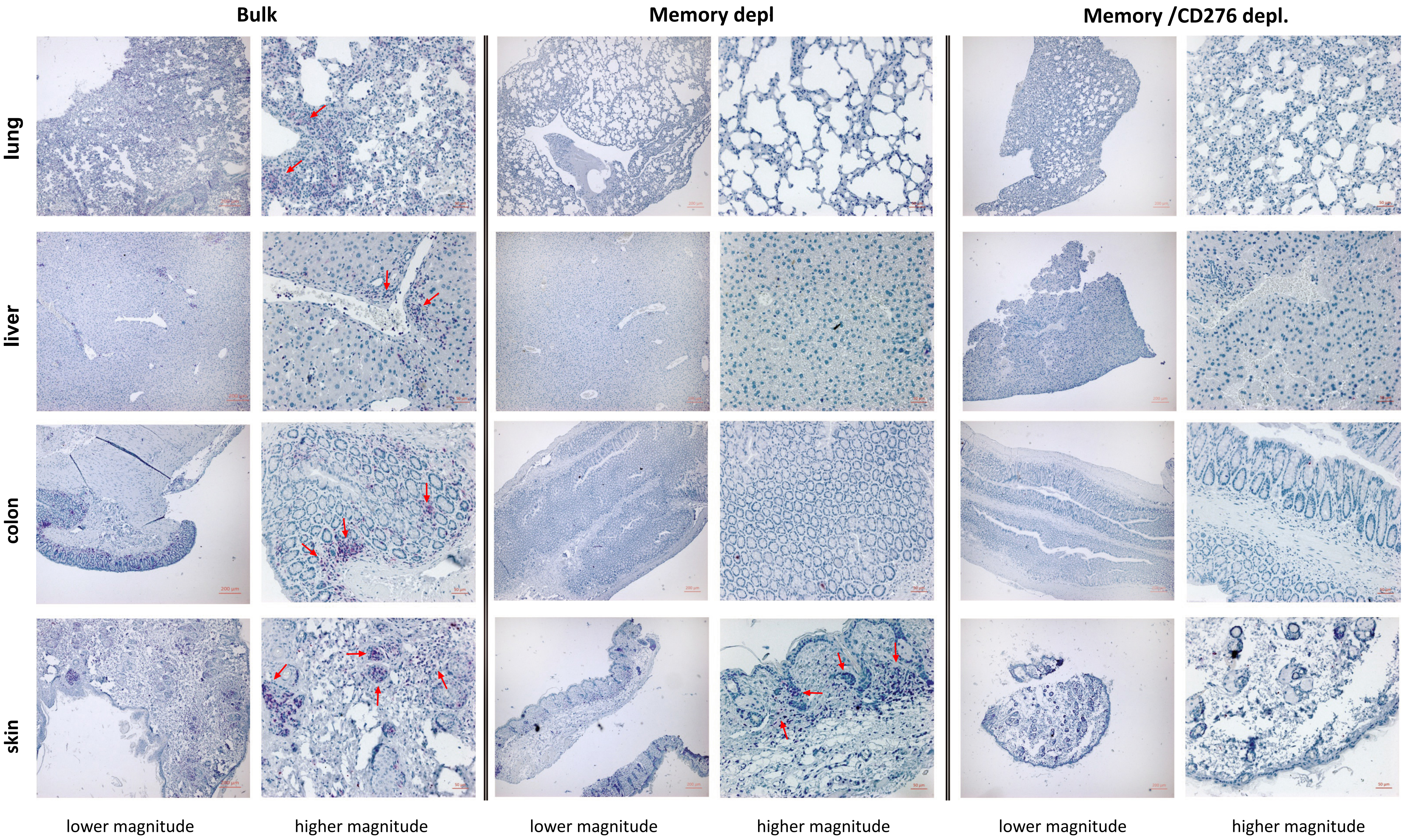

Supplementary Figure 10: Histological analysis of GVHD target organs in DR4-matched transplant. Tissues from NSG-Ab° DR4 mice were fixed in 10% buffered formalin and embedded in paraffin for hematoxylin and eosin (H&E) staining and immunohistochemical staining with anti-human CD3.

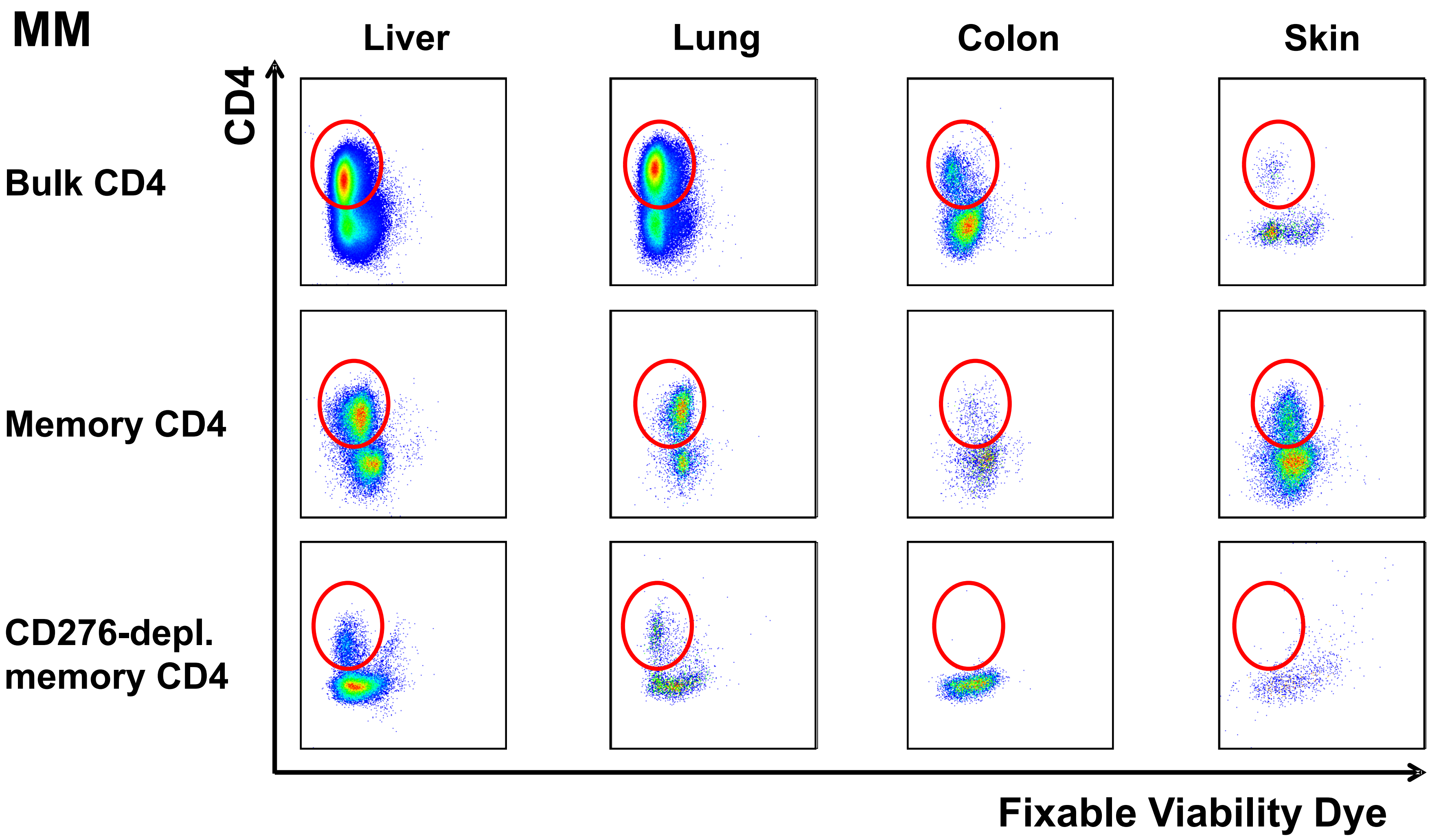

Supplementary Figure 11: GVHD target organ infiltrating T cells were analyzed by flow cytometry. Resected tissue specimens were disaggregated into single cell suspensions, and stained for flow cytometric analysis. Representative flow cytometry plots depict target organ infiltrating T cells ($CD4^{+}$ Fixable viability^{-}) in red circles.

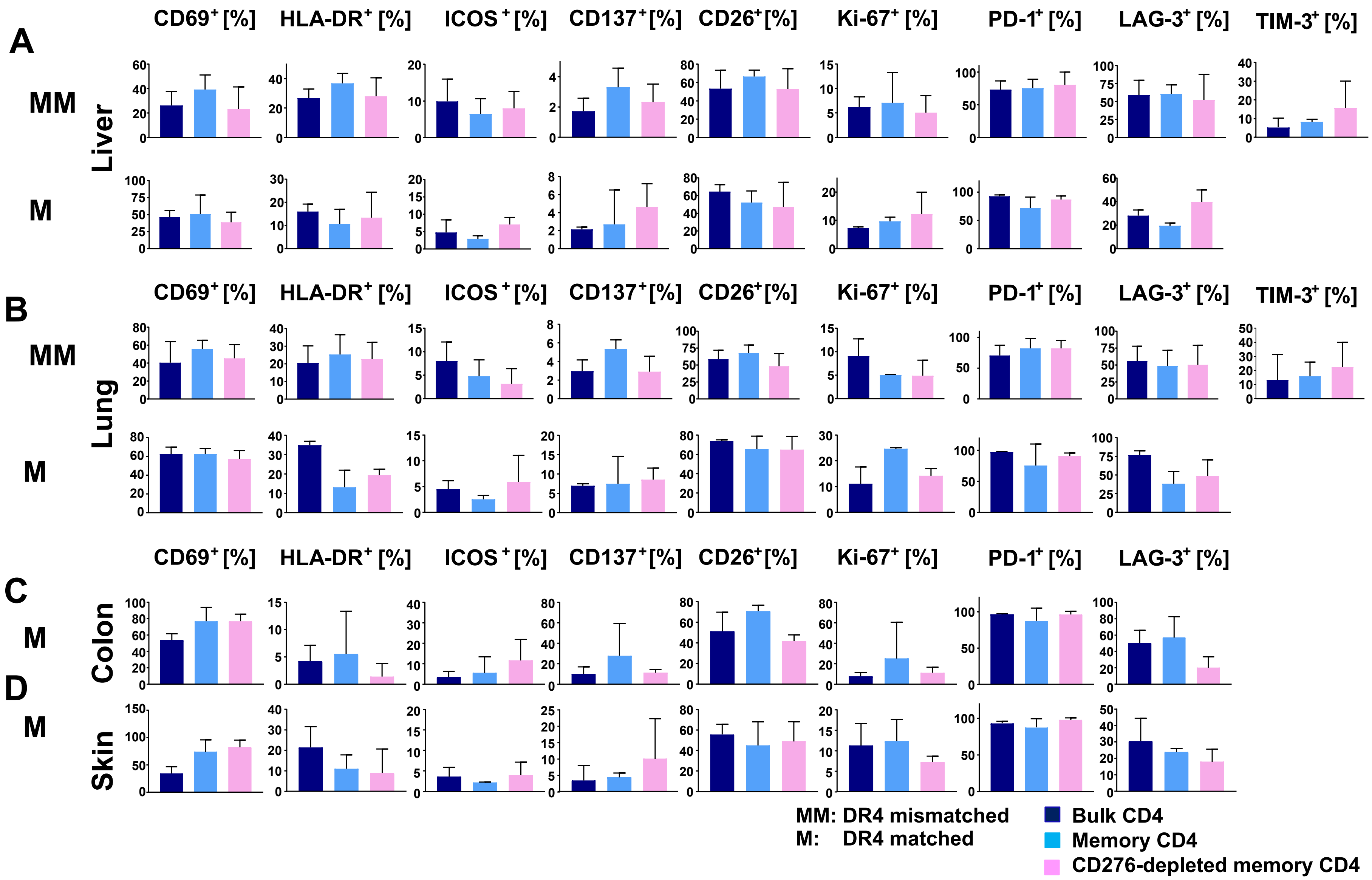

Supplementary Figure 12: Activation status of GVHD target organ infiltrating T cells. (A) Liver-infiltrating human $CD4^+$ T cells, and (B) Lung-infiltrating human $CD4^+$ T cells from DR4 mice, (C) Colon-infiltrating human $CD4^+$ T cells, (D) Skin-infiltrating human $CD4^+$ T cells were analyzed when mice were sacrificed. In DR4-mismatched setting (MM): bulk $CD4^+$ T cells n=5, memory $CD4^+$ T cells n=4, CD276-depleted memory $CD4^+$ T cells n=4. In DR4-matched setting (M): bulk $CD4^+$ T cells n=5, memory $CD4^+$ T cells n=3, CD276-depleted memory $CD4^+$ T cells n=4.

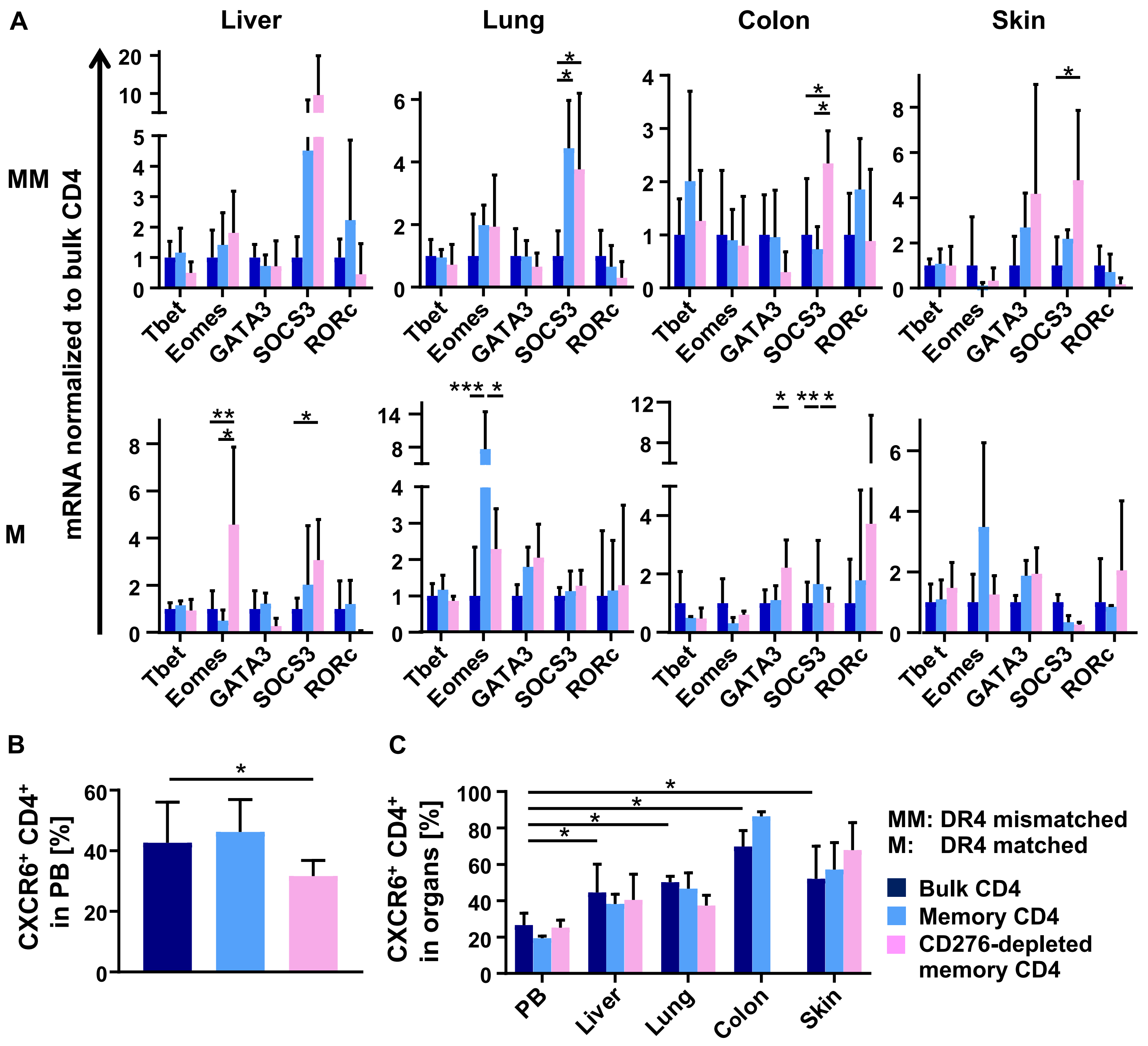

Supplementary Figure 13: Transcription factors and CXCR6 expression of organ-infiltrating and peripheral T cells. (A) Transcription factors expressed in target organ infiltrating T cells were analyzed by qPCR when mice were sacrificed. (B) CXCR6 expression on peripheral $CD4^+$ T cells was examined when DR4 mice had achieved human $CD4^+$ T cells engraftment. (C) CXCR6 expression of organ-infiltrating $CD4^+$ T cells was examined when mice were sacrificed. $^*P < 0.05$, $^{**}P < 0.01$, $^{***}P < 0.001$ by one-way ANOVA followed by Tukey's multiple comparison test.

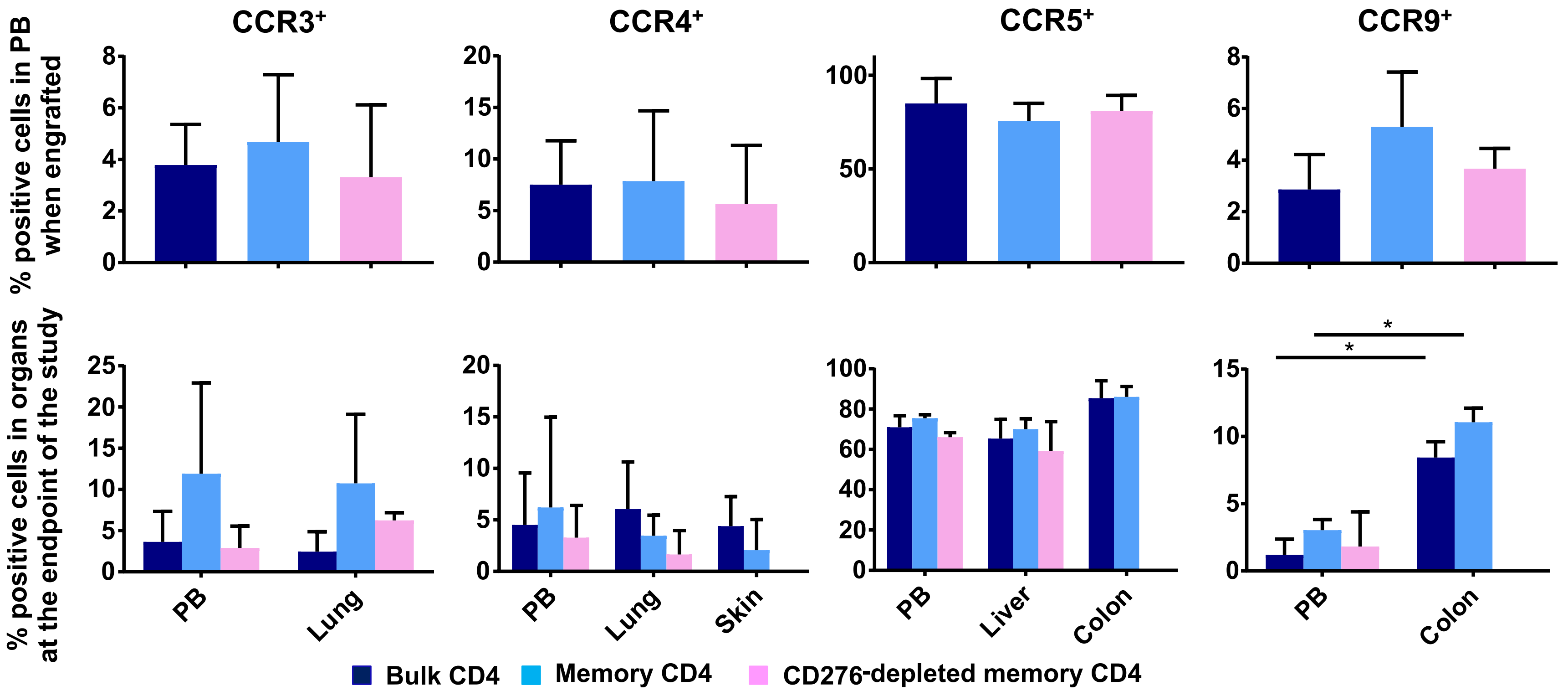

Supplementary Figure 14: Chemokine receptor expression of engrafted human cells. Colon-infiltrating T cells show a higher level of CCR9 than peripheral T cells, CCR9 expression on circulating T cells at engraftment was similar in all cohorts. The expression of the chemokine receptors CCR3, CCR4, CCR5 was not upregulated in organs and expression levels on circulating T cells at engraftment and did not differ between cohorts. *P < 0.05 by one-way ANOVA followed by Tukey's multiple comparison test.

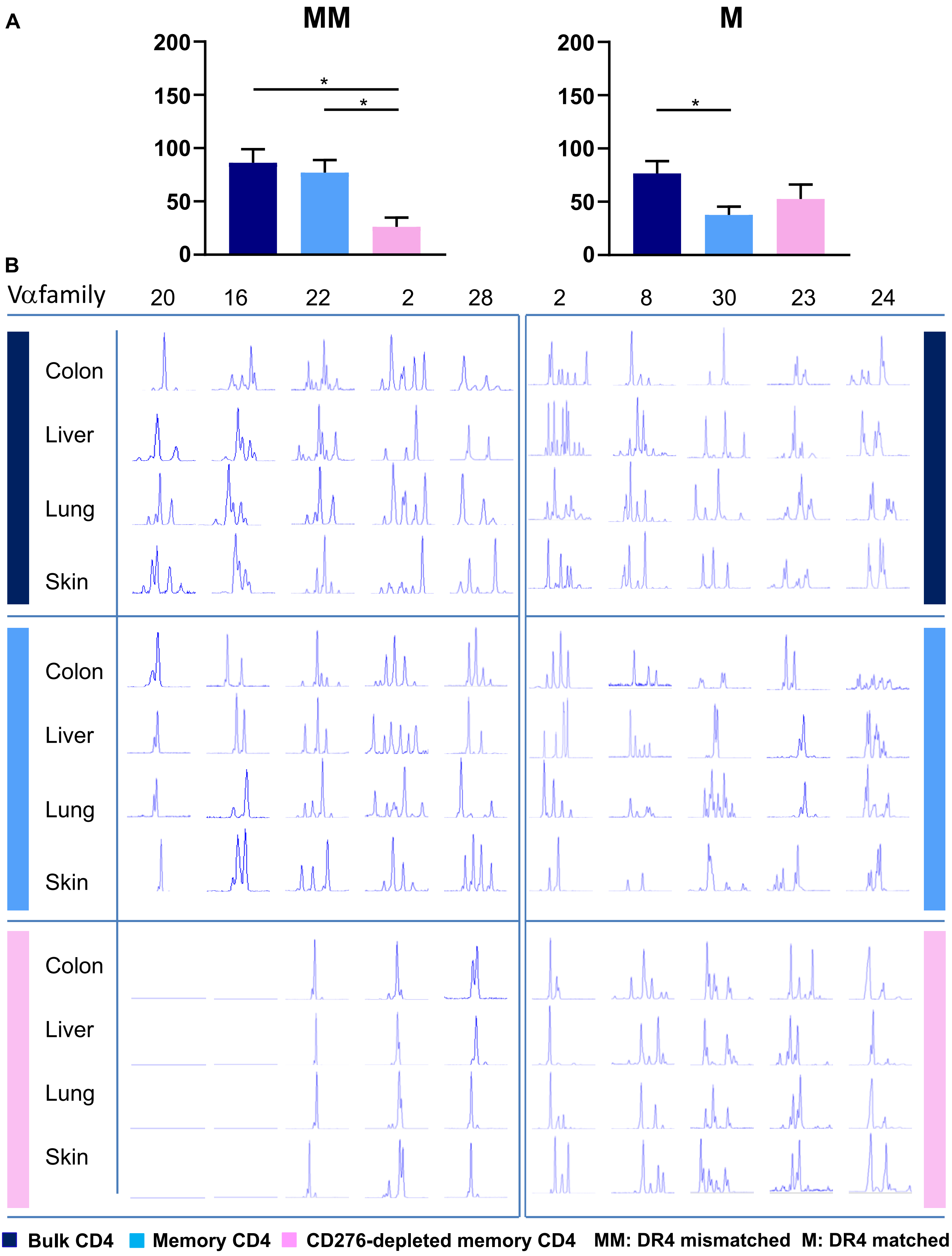

Supplementary Figure 15: Reduction of complexity scores of TCR Vα chains in GVHD target organ infiltrating T cells by CD45RA/CD276-depletion in DR4-mismatched transplant. (A) The overall TCR complexity score for 34 Vα gene families was calculated for GVHD target tissues from NSG-Ab° DR4 mice. Mean + SD of TCR complexity scores in a single organ per mouse are shown. (B) Representative TCR Vα spectratypes of target organ-infiltrating T cells transplanted with differently engineered grafts with all grafts generated from the same donor. CDR3 length versus fluorescence intensity is presented on the x-axis and the y-axis respectively. *P < 0.05 by one-way ANOVA followed by Tukey's multiple comparison test.

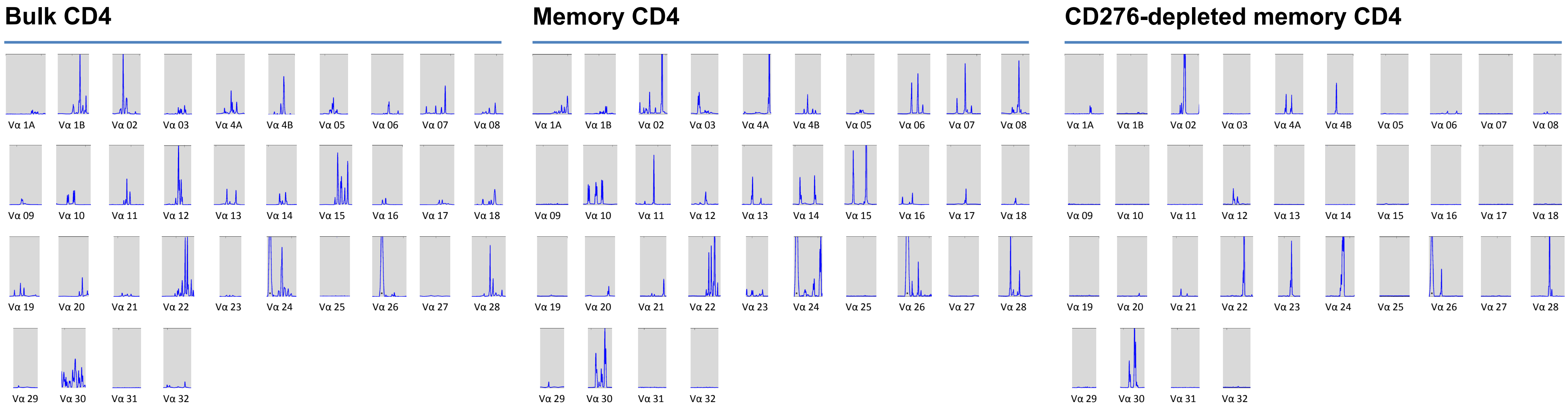

Supplementary Figure 16: Vα-chains Gaussian distribution pattern skewed to single peaks indicate CD45RA/CD276-depletion reduces the repertoire diversity of alloreactive T cell pools in DR4-mismatched transplantation. The repertoire of 24 different TCR Vα-chains of GVHD target organ infiltrating T cells was analyzed by TCR spectratyping in all cohorts. Representative data of liver-infiltrating T cells from a DR4^{negative} donor are shown.

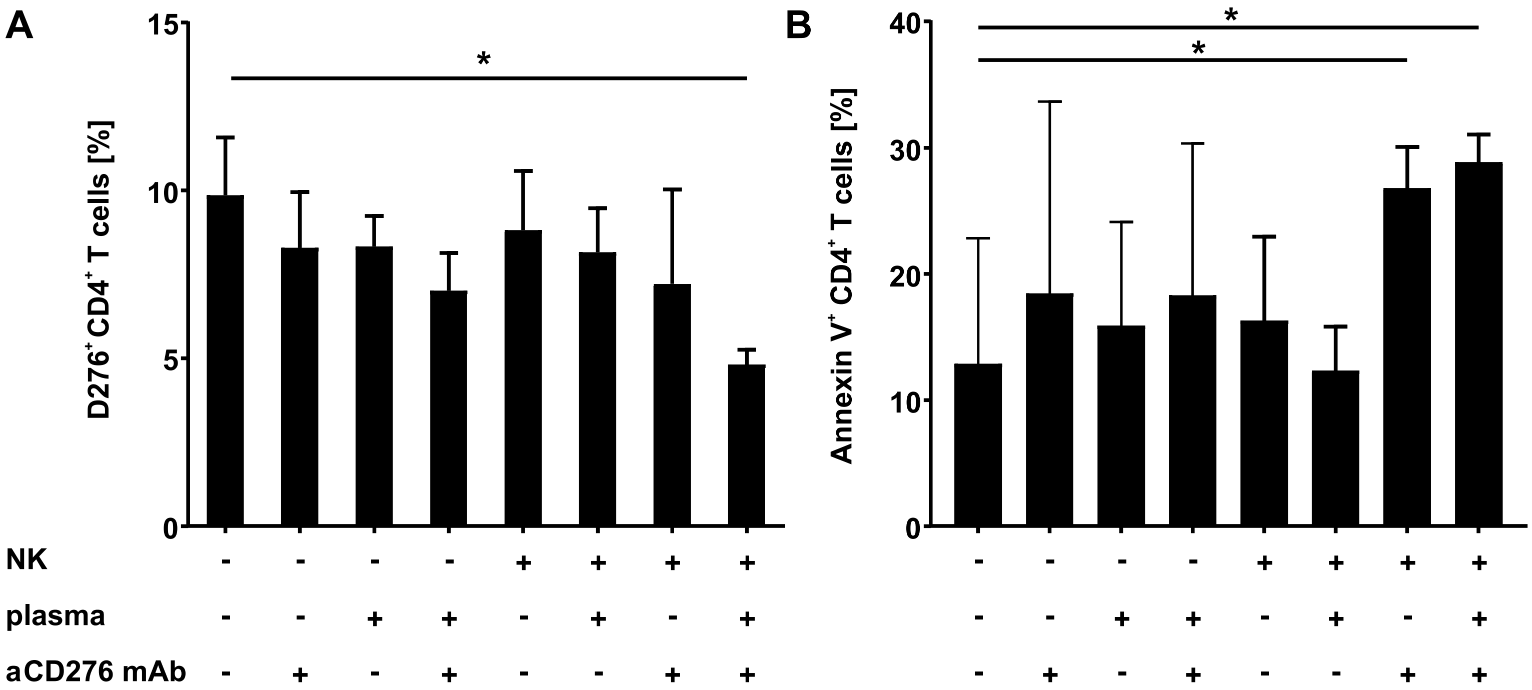

Supplementary Figure 17: The anti-CD276 mAb exhibits ADCC activity *in vitro*. $CD4^+$ T cells were isolated from PBMCs and incubated with anti-CD276 depleting mAb in the presence or absence of autologous NK cells/autologous plasma for 24 hours at 37 °C. Cells were harvested and apoptotic $CD4^+$ T cells were analyzed by staining Annexin-V and 7-AAD. The frequency of $CD276^+$ T cells in $CD4^+$ T cells after 24 hour-incubation is shown on the left and the frequency of Annexin-V^+ apoptotic cells in $CD4^+$ T cells after 24 hour-incubation is shown on the right (n=3). *$P < 0.05$ by one-way ANOVA followed by Tukey's multiple comparison test.

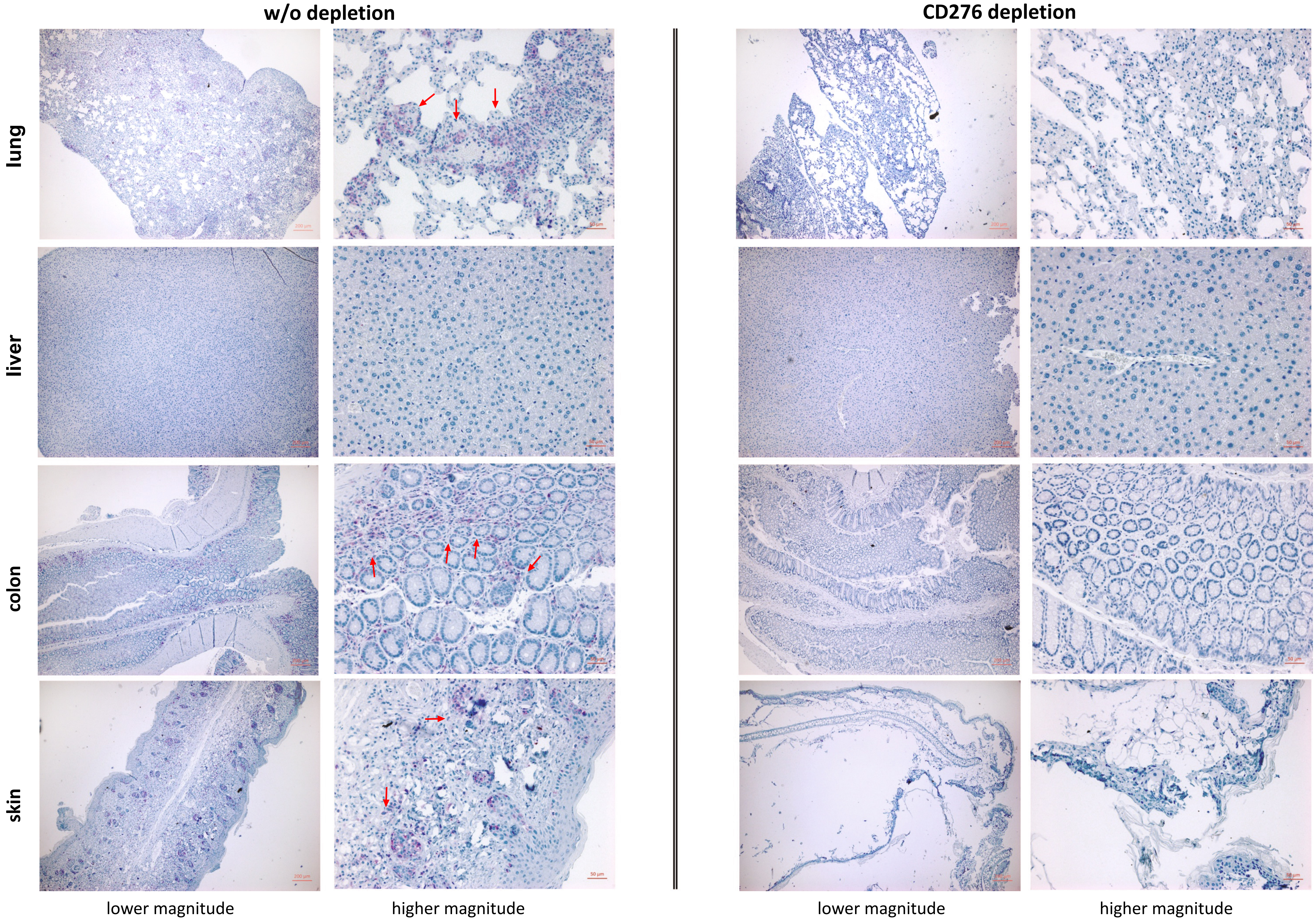

Supplementary Figure 18: Histological analysis of GVHD target organs for infiltrating CD3 T cells in controls or after in vivo CD276-depletion. Tissues from NSG-Ab° DR4 mice were fixed in 10% buffered formalin and embedded in paraffin for hematoxylin and eosin (H&E) staining and immunohistochemical staining with anti-human CD3.

Table S1: The primer sequences for qPCRs used in this study

GAPDH	Forward	5'-CCACATCGCTCAGACACCAT-3'
	Reverse	5'-GGCAACAATATCCACTTTACCAGACT-3'
T-bet	Forward	5'-GCCTACCAGAATGCCGAGATTA -3'
	Reverse	5'-ACTCAAAGTTCTCCCGGAATCC-3'
RORC	Forward	5'-CAGTGAGAGCCCAGAAGGAC-3'
	Reverse	5'-TCTTGGCCTTCATTGTACCC -3'
Foxp3	Forward	5'-GAGAAGCTGAGTGCCATGCA-3'
	Reverse	5'-GGAGCCCTTGTCGGATGAT-3'
Gata3	Forward	5'-GCGGGCTCTATCACAAAATGA-3'
	Reverse	5'-GCCTTCGCTTGGGCTTAAT-3'
Eomes	Forward	5'- GGCAAAGCGGACAATAACAT-3'
	Reverse	5'-AGCCTCGGTTGGTATTTGTG-3'

Table S2: Antibodies used for flow cytometry in this study

specificity	isotype	clone	manufacturer	catalogue number	fluoroscence
CD28	Mouse IgG_1, κ	CD28.2	BD Biosciences	612815	BUV737
PD-1	Mouse IgG_1, κ	EH12.1	BD Biosciences	612791	BUV737
HLA-DR	Mouse IgG2a, κ	G46-6	BD Biosciences	612981	BUV661
CD25	Mouse IgG_1, κ	M-A251	BD Biosciences	740290	BUV395
ICOS	Mouse IgG2b, κ	2D3/B7-H2	BD Biosciences	743011	BUV395
CD161	Mouse IgG_1, κ	DX12	BD Biosciences	744096	BV786
CD276	Mouse IgG_1, κ	7-517	BD Biosciences	565829	BV421
CCR6	Mouse IgG_1, κ	11A9	BD Biosciences	551773	PE
CCR4	Mouse IgG_1, κ	1G1	BD Biosciences	551120	PE
CD45RA	Mouse IgG_1, κ	5H9	BD Biosciences	561216	PE-Cy7
CCR7	Mouse IgG_1, κ	2-L1-A	BD Biosciences	566769	PE-CF594
CD26	Mouse IgG_1, κ	M-A261	BD Biosciences	565158	PE-CF594
CD95	Mouse IgG_1, κ	DX2	BD Biosciences	556640	FITC
CD3	Mouse IgG_1, κ	UCHT1	BD Biosciences	555332	FITC
CD45	Mouse IgG_1, κ	HI30	BD Biosciences	561864	APC
CD8	Mouse IgG_1, κ	HIT8α	BD Biosciences	566855	APC-H7
LAG-3	Mouse IgG_1, κ	11C3C65	BioLegend	369314	BV421
CD3	Mouse IgG2a, κ	HIT3α	BioLegend	300324	Alexa700
IL-17A	Mouse IgG_1, κ	BL168	BioLegend	512310	Alexa647
CD69	Mouse IgG_1, κ	FN50	BioLegend	310910	APC
Ki-67	Mouse IgG_1, κ	Ki-67	BioLegend	350526	PE-Cy7
CXCR6	Mouse IgG2a, κ	K041E5	BioLegend	356012	PE-Cy7
CD8	Mouse IgG_1, κ	HIT8α	BioLegend	300922	PerCP
CD4	Mouse IgG_1, κ	SK3	BioLegend	344624	PerCP
TNF-α	Mouse IgG_1, κ	MAb11	BioLegend	502948	BV785
IFN-γ	Mouse IgG_1, κ	B27	BioLegend	506507	PE
TIM-3	Mouse IgG_1, κ	F38-2E2	ThermoFisher Scientific	17-3109-42	APC
CD137	human IgG1	REA765	Miltenyi Biotech	130-110-765	FITC
CCR3	human IgG1	REA574	Miltenyi Biotech	130-108-890	APC
CCR5	human IgG1	REA245	Miltenyi Biotech	130-120-057	APC
CD4	human IgG1	REA623	Miltenyi Biotech	130-114-534	Vioblue
CCR9	Mouse IgG2a	112509	R&D	MAB179-100	FITC

Table S3: TRAV CDR3 sequences of GVHD target infiltrating T cells. TCR epitope-binding region (CDR3) sequences derived from single peaks in TCR Vα-repertoire spectratype analysis of GVHD target organ infiltrating T cells were identified in direct sequencing approaches and amino acid sequences were delineated. TCRα binding regions (CDR3 regions) identified in recipients of one of the three different graft types, all grafts generated from the same donor, are shown for a representative HLA-DR4^{negative} (MM) and a DR4^{positive} (M) donor.

	MM								M							
	Organ	TRAV	TRAJ	Variable	N	Joining	public motif	Reactivity	Organ	TRAV	TRAJ	Variable	N	Joining	public motif	Reactivity
bulk CD4	Liver	14	56	CA	MRAP	GANSKLTF GKG			Liver	26	31	CI	GRCDNA	ARLMFGDG	public	Influenza A
	Liver	26	39	CAL	VGAISRILTLAWTSKKT	FGKG			Liver	2	30	CAV	DRDDNPSFLWKRDT	FGYG		
	Lung	22	49	CA	GPGYSG	YFGYG			Liver	39	13	CAV	TN	GGYQKVTFGIG	public	CMV
	Lung	26	39	CIV	RVG	NNAGNMLTFGGG	public	Influenza A	Liver	26	47	CI	L RDN	FGFG		
	Lung	23	9	CAAS	TRLRRDQIVALHQG	FGAG			Liver	25	36	CA	germline	QTGANNLFFGTG	public	CMV
	Colon	22	49	CAV	AA	NTGNQ FYFGTG	public	CMV	Lung	22	40	CAL	S	TSGTYKYIFGTG		
	Skin	26	52	CA	RKSHR	FGKG			Lung	26	31	CI	GIDRFT	FGVG		
	Skin	22	49	CAV	AGYSGYGIS	FGTG			Lung	25	36	CA	GTGAHT	LFFGTG		
	Skin	14	56	CA	SESAP	GANSKLTFGKG			Lung	26	56	CIV	germline	TF GIG		
	Skin	3	32	CAV	RGVNGE	KLIFGGG			Lung	3	5	CAV	RDSYTDS	RALTFGSG		
									Lung	39	14	CIS	RTPRQGQ	FGSG		
									Colon	26	3	CAS	germline	KIIFGSG		
									Skin	25	9	PAS	FSLQKKRGG	FGAG		
									Skin	38	52	CA	LSG	FGAG		
									Skin	2	36	CA	EGGKTT	FGKG		
									Skin	26	2	CIA	EL	GIG		
									Skin	3	15	CAV	RLT	N QAGTALIFGKG	public	CMV
memory CD4	Liver	2	37	CAV	EAG	NTGKLIFGQG			Liver	19	2	CAL	SGDNQGG	KLTFGLG		
	Liver	10	6	CAV	IELGI	SGGSYIPTFGRG			Liver	2	18	CAV	GSGSAQ	GSG		
	Lung	23	44	CA	PGTNSAGGG	LTFGTG			Liver	25	53		CRVGVIVGG	FGEG		
	Lung	26	53	CI	LRDPDR	GGSNYKLTFGKG			Lung	9-2	12	CI	RGLSVCVMGSKGL	IFGSG		
	Lung	2	37	CAV	EAG	NTGKLIFGQG			Skin	3	8	CAV	RDMG	TGFQKLVFGTG		
	Lung	29	52	CA	AGSG	AGGTSYGKLTFGQG	public	Insulin	Skin	25	40	CA	AI	TSGTYKYIFGTG	public	Influenza A
	Colon	8-3	18	CA	GTR	FGQG			Skin	2	20	CAL	FIAAHP	FGAG		
	Colon	29	52	CA	ST	AGGTSYGKLTFGQG			Skin	24	20	CAF	LCPLLWDDYK	FGAG		
	Colon	13-1	20	CA	ASVG	SNDYKLSFGAG	public	CMV	Skin	5	29	CA	EGH	SGNTPLVFGKG		
	Skin	29	18	CAV	QFKI	FGQG										
	Skin	2	37	CAV	EAG	NTGKLIFGQG										
CD276-depleted memory CD4	Liver	12	25	CAV	RGEYETPCDCTR	FGRG			Liver	8-4	20	CA	SPLRCRTRVDHE	FGAG		
	Liver	20	37	CAV	WRGTTQA	KLIFGQG			Liver	5	7	CAV	CDKLII	FGEG		
	Liver	1	32	CAV	SGRR	GATNKLIFGTG	public	CMV	Lung	30	14	CA	FSLEY	FGGG		
	Lung	25	54	CAS	L	IQGAQKLVFGQG			Lung	9-2	12	CI	RGLSVCVMGSKGL	IFGSG		
	Lung	26-1	23	CI	VRS	NQGGKLIFGQG			Colon	23	11	CA	TKRFSSAPQL	FGKG		
	Lung	9-2	6	CI	DLNDT	GGSYIPTFGRG			Skin	24	3	CA	P	YSSASKIIFGKG	public	EBV
	Colon	3	5	CAV	RDGNTGRRAL	TFGSG			Skin	26	42	CIV	CHTR	GSQGNLIFGKG		
	Colon	26-1	42	CI	VCPLEEATVISP	FGKG			Skin	26	53	CAV	VHSSISQGSTVQDQ	GTG		
	Skin	3	5	CAV	RDGNTGRRAL	TFGSG			Skin	8-3	6	CAV	GAR	GSYIPTFGRG	public	CMV
	Skin	38	43	CAV	LIGAL	MRFGAG			Skin	8-4	15	CAV	RAF	NQAGTALIFGKG		
	Skin	9-2	6	CA	LSET	GGSYIPTFGRG	public	CMV								
	Skin	8-4	33	CAV	SDRII	YQLIWGAG	public	CMV								
